# Estimation of the Optimal Statistical Quality Control Sampling Time Intervals Using a Residual Risk Measure

**DOI:** 10.1371/journal.pone.0005770

**Published:** 2009-06-09

**Authors:** Aristides T. Hatjimihail

**Affiliations:** Hellenic Complex Systems Laboratory, Drama, Greece; University of East Piedmont, Italy

## Abstract

**Background:**

An open problem in clinical chemistry is the estimation of the optimal sampling time intervals for the application of statistical quality control (QC) procedures that are based on the measurement of control materials. This is a probabilistic risk assessment problem that requires reliability analysis of the analytical system, and the estimation of the risk caused by the measurement error.

**Methodology/Principal Findings:**

Assuming that the states of the analytical system are the reliability state, the maintenance state, the critical-failure modes and their combinations, we can define risk functions based on the mean time of the states, their measurement error and the medically acceptable measurement error. Consequently, a residual risk measure *rr* can be defined for each sampling time interval. The *rr* depends on the state probability vectors of the analytical system, the state transition probability matrices before and after each application of the QC procedure and the state mean time matrices. As optimal sampling time intervals can be defined those minimizing a QC related cost measure while the *rr* is acceptable. I developed an algorithm that estimates the *rr* for any QC sampling time interval of a QC procedure applied to analytical systems with an arbitrary number of critical-failure modes, assuming any failure time and measurement error probability density function for each mode. Furthermore, given the acceptable *rr*, it can estimate the optimal QC sampling time intervals.

**Conclusions/Significance:**

It is possible to rationally estimate the optimal QC sampling time intervals of an analytical system to sustain an acceptable residual risk with the minimum QC related cost. For the optimization the reliability analysis of the analytical system and the risk analysis of the measurement error are needed.

## Introduction

In clinical chemistry the minimal required frequency of statistical QC applied to analytical systems has been the testing of at least one control sample per level of concentration of the measurand, at two levels of concentration, once per 24 hr. A few years ago the Clinical Laboratory Improvement Amendments (CLIA) recommendation of the “equivalent QC” was issued [1]. According to that recommendation the control materials based QC that is not built in the test system but is applied by the user is characterized as “external” QC, while the “internal monitoring systems that are a part of or built into the test system are called electronic, internal, or procedural controls”. The required “external” QC frequency can be reduced to once per month or once per week, after an initial evaluation period of successful daily QC testing. A system to be eligible for the QC frequency of once per month should include an internal monitoring system. The “equivalent QC” concept has implicitly introduced the reliability concept of the critical-failure rate into QC planning. The system is evaluated by daily QC testing for a certain period of time. If the evaluation is successful then it is assumed a critical hazard rate less than an implicit threshold and a less frequent QC is required. On the other hand less frequent QC is required if the system includes an internal monitoring system, as this system detects the potential critical failures, except if it fails too. In that case the hazard rate of the system is the rate of a combined potential critical failure of the analytical subsystem and of the internal monitoring system. The equivalent QC concept has initiated a debate about the optimal QC frequency.

There have been proposed optimization methods of the QC procedures applied to clinical chemistry analytical systems [2]–[4]. They maximize the probability of rejection, assuming that certain significant measurement error has been introduced into the measurements, while keeping the probability for false rejection less than a certain threshold. There also have been references to the run length. In their paper, Westgard, Koch and Oryall [5] used the batch size and the observed frequency of errors to estimate the test yield and defect rate. To estimate the test yield they proposed an expanded productivity model that included an estimation of the repetition of the tests because of the erroneous results. Parvin and Gronowski proposed performance measures based on the analytical run length and the number of patient samples with unacceptable error [6]. Parvin and Robbins estimated the mean time “from the occurrence of an out-of-control error condition to the next scheduled QC event”, assuming an exponential distribution of failure, to compare the performance of randomized versus fixed-time schedules of QC procedures [7]. Recently, Parvin used a worst case of measurement error scenario estimation of the number of the samples nonconforming the quality specifications to propose a definition of the sampling time interval [8]. Nevertheless, in clinical chemistry the estimation of the optimal sampling time intervals for the application of statistical quality control (QC) procedures that are based on the measurement of control materials remains an open problem.

In the QC literature there have been papers on the economic design of the 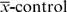 charts, optimizing the number of the controls, the decision limits and the sampling time interval to minimize the cost. Duncan proposed a fixed run length optimization method, assuming one failure mode with an exponential distribution of failures [9], while Banerjee and Rahim proposed an elegant variable run length optimization method, assuming one failure mode with a Weibull distribution of failures [10]. In addition they optimized the number of the controls and the decision limits to minimize the cost of the production process, including the QC cost. Linderman, McKone-Sweet, and Anderson [11], and recently Panagiotidou and Nenes [12] have proposed an integrated approach to process control and maintenance.

The explosive growth of the complexity of the clinical laboratories and of their analytical systems increases exponentially the difficulty of their management. New quantitative tools are needed to assist the sound judgment of their directors so that they can take optimal or near optimal decisions. We are actually experiencing a paradigm shift in the management of the clinical laboratories, as new techniques are introduced from other fields. Particularly promising are the risk management techniques that have recently been applied in some clinical laboratories, although they have already been extensively and successfully used in engineering.

As *risk management* is defined “the systematic application of management policies, procedures, and practices to the tasks of analyzing, evaluating, controlling, and monitoring risk” [13]. *Risk* is “the combination of the probability of occurrence of harm and the severity of that harm” while *hazard* is “the potential source of harm”. *Residual risk* is the risk remaining after the control measures have been taken. To be applied to the analytical systems risk management needs to be supplemented by reliability analysis. *Reliability* is “the probability that an item will perform a required function, under stated conditions, for a stated period of time”. Reliability is therefore *the extension of quality into the time domain* and may be paraphrased as “the probability of non-failure in a given period” [14]. *Failure* is the “termination of the ability of an item to perform a required function”, while *critical failure* is “a failure that can initiate hazard”.

Actually, the QC planning problem of the analytical process can be translated into a probabilistic risk assessment problem. The reliability analysis of an analytical system should include a quantitative fault tree analysis [15] to define the critical-failure modes and estimate the critical-failure time and measurement error probability density functions and their dependencies. A critical failure of an analytical system in a clinical laboratory setting can initiate hazard when the total measurement error of a result of a patient exceeds the medically acceptable measurement error. This incorrect result can cause harmful medical decisions. The risk of a critical failure is associated with the probability that it will occur and with the time that it will persist. The applied QC procedure detects a critical failure with a certain probability. As residual risk can be considered the risk of the measurement process, assuming the application of the QC procedure. We can define risk measures based on the partial moments of the measurement error with reference to the medically acceptable measurement error (see *Partial moments* in Appendix S1). Then we can estimate the risk before the application of the QC and the residual risk assuming QC is applied.

There is a certain financial cost associated with the QC, including the cost of the control materials and their measurements and the cost of the repetitions because of the rejections. Therefore, an operational approach to the optimal QC sampling planning could be based on the minimization of the QC related cost while the residual risk is acceptable.

To explore the estimation of the QC sampling time intervals using a residual risk measure I developed an algorithm that estimates the residual risk of any sampling time interval of QC procedures applied to analytical systems with an arbitrary number of critical-failure modes, assuming any probability density function of critical-failure time and measurement error for each mode. Furthermore it can estimate the optimal QC sampling time intervals that minimize a QC related cost measure.

## Methods

The Mathematica® 7.0 mathematical program was used for the development of the algorithm. A personal computer with an Intel Quad Core® 2.8 GHertz CPU, 8 GBytes of RAM, and the 64-bit Windows Vista® operating system was used for the estimation of the data.

Using advanced numerical methods the algorithm I developed estimates the residual risk of any sampling time interval of a QC procedure applied to analytical systems with an arbitrary number of critical-failure modes, assuming any probability density function of critical-failure time and measurement error for each mode. Furthermore it can estimate the optimal QC sampling time intervals [*t_i−1_*,*t_i_*] to minimize a QC related cost measure, given the maximum acceptable residual risk measure *rr*.

### The model

The algorithm is based on a model that simulates a clinical analytical system. The main components and parameters of the model are:

1. The medically acceptable measurement error.
2. The states *S* of the model:
  - The *n* critical-failure modes *F_h_* and their combinations. The critical-failure modes can be independent or dependent upon each other.
  - The reliability state *R*.
  - The maintenance state *M*.
3. The critical-failure time probability density functions f*_h_* of each critical-failure mode *F_h_*.
4. The measurement error probability density functions:
  - The measurement error probability density function g_0_ of the reliability state *R*.
  - The measurement error probability density function g*_h_* of each critical-failure mode *F_h_*.
5. The initial time *t*_0_ and the initial state probability vector **p**_0_(**s** *_n_*) of the system (see eqs (41) and (42)).
6. The series of the measurements performed at each QC sampling time interval (*t_i−1_*,*t_i_*).
7. The applied QC procedure:
  - The *c* levels of the concentration of the measurand of the controls
  - The number of the controls at each level
  - The QC rules
8. The risk functions of the states.
9. The QC related cost functions of the states.

### Definitions

The definitions of the functions, vectors, matrices and equations (see *Notation* in Appendix S1) used for the simulations are given with the following additional assumptions:

1. For *t*_0_ = 0 the initial state of the system is the reliability state *R*.
2. The algorithm is applied until the system enters the maintenance state.
3. There are one or two critical-failure modes *F_h_*.
4. The critical-failure time probability density functions f*_h_* of each critical-failure mode are general distributions.
5. The critical-failure modes and their time probability density functions f*_h_* are independent.
6. The probability density function *g*_0_ of the reliability state is the unit normal distribution. Therefore, *μ*_0_ = 0 and *σ*_0_ = 1, where *μ*_0_ and *σ*_0_ are the mean and the standard deviation of the *g*_0_. The probability density function *g_h_* of the measurement error of each critical-failure mode is either the normal distribution or a mixture distribution that models the intermittent measurement error. If the system fails with two combined failure modes, then an additive measurement error model is assumed.
7. The probability density functions of the measurement error at each level of the controls are the same. If the probability density functions of the measurement error of the *c* levels of the concentration of the measurand of the controls are different, then multivariate probability density functions can be used (See *Definition of functions assuming multivariate measurement error probability density functions* in Appendix S1).
8. The QC rules are single value rules with decision limit *l* [16], applied at the end of the QC sampling time interval upon one control per level at *c* levels of controls.
9. The rejection of an analytical run by the QC procedure causes the transition of the analytical system to the maintenance state.
10. The measurement error, the critical measurement error, the medically acceptable measurement error, and the decision levels *l* of the controls are measured in *σ*_0_ units, where *σ*_0_ is the standard deviation of the measurement error of the reliability state.
11. The time is measured in arbitrary time units.
12. The cost is measured in cost per unit of time of operation in the reliability state units.
13. The risk of the states is a function of the *d* ^th^ partial moments of their measurement error with reference to the medically acceptable measurement error.
14. The QC related cost of the states for a sampling time interval is the cost *cq* of the *c* controls, where *q* is the cost of each control sample and its mesurement. In addition, the QC related cost of the maintenance state includes a cost *m* and the cost of the repetitions of the analysis of the controls and the samples.

### Definition of the functions

#### Reliability functions

The analytical systems fail with a certain probability during their lifetime. Usually there are several failure modes. Assuming that f*_j_(t)* is the failure time probability density function of the *j* ^th^ failure mode, the respective mean time to failure is defined as
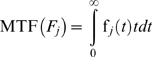
(1)while the hazard rate is defined as
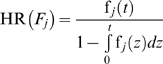
(2)We may assume that
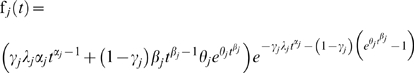
(3)where *α_j_*, *β_j_*, *θ_j_*, and *λ_j_*≥0 and 0≤*γ_j_*≤1. This distribution is called general distribution [17].

**Figure 1:**
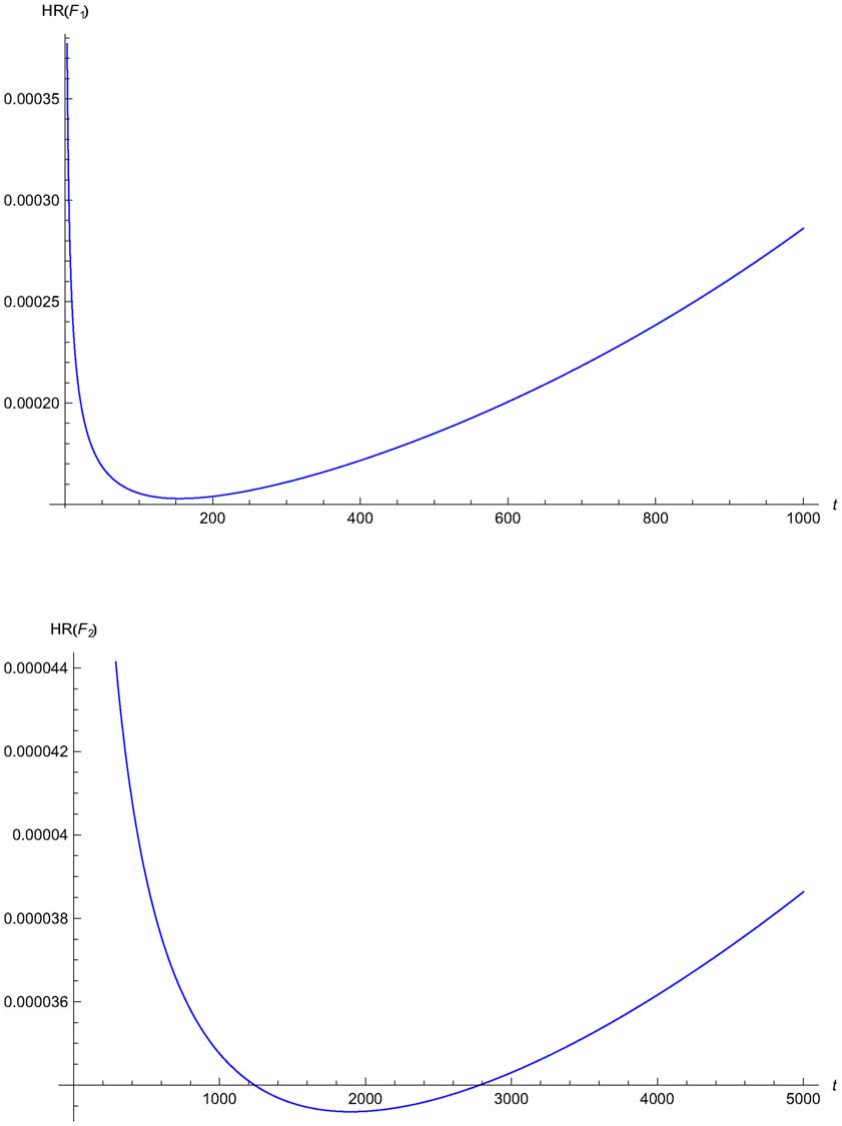
Bathtub hazard rate curves. Upper plot: The hazard rate HR(*F_1_*) of the general failure time probability density function for *α*_1_ = 0.5, *β*_1_ = 1, *γ*_1_ = 0.9, *θ*_1_ = 0.001, and *λ*_1_ = 0.001, with a bathtub curve. Lower plot: The hazard rate HR(*F_2_*) of the general failure time probability density function for *α*_2_ = 0.5, *β*_2_ = 1, *γ_2_* = 0.8, *θ*_2_ = 0.0001, and *λ*_2_ = 0.001, with a bathtub curve.

For *α_j_* = 1 and *γ_j_* = 1, we have the exponential distribution:
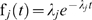
(4)while i.e, for *α_j_* = 0.5 and *β_j_* = 1 we have a distribution with a bathtub hazard rate curve (figure 1).

The probability that the system fails at the *i* ^th^ time interval because of the *j* ^th^ mode is:
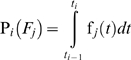
(5)


The conditional probability that the system fails at the *i* ^th^ time interval because of the *j* ^th^ mode, given that it has not failed because of this failure mode at the time *t_i−1_* is:
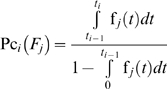
(6)


The probability that the system fails at the *i* ^th^ sampling time interval because of the *j* ^th^ and *h* ^th^ failure modes is:
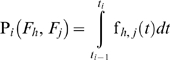
(7)


Assuming that the two failure modes are independent, we have:
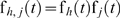
(8)


Therefore, the conditional probability that the system fails at the same sampling time interval because of the *j* ^th^ and *h* ^th^ failure modes, given that it has not failed because of these failure modes at the time *t_i−1_* is:
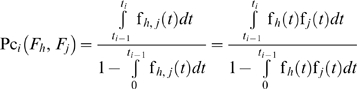
(9)


The conditional probability that the system fails at the *i* ^th^ sampling time interval because of the *h* ^th^ failure mode, given that it has failed because of the *j* ^th^ failure mode before the *i* ^th^ sampling time interval, is:
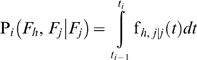
(10)


Assuming that the failure time probability density functions of the two failure modes are independent, we have:
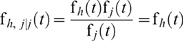
(11)Therefore:
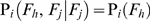
(12)and
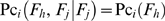
(13)


If the failure time probability density functions of the two failure modes are dependent, we have
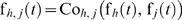
(14)and
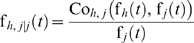
(15)where Co*_h,j_*(f*_h_*(*t*), f*_h_*(*t*)) is a bivariate dependence function.

The expected time from the failure of a system because of the *j* ^th^ failure mode until the time *t_i_*, given that the failure has happened at the *i* ^th^ sampling time interval is:
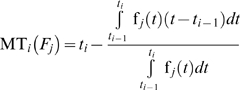
(16)


The expected time from the failure of a system because of the *j* ^th^ and *h* ^th^ failure modes until the time *t_i_*, given that the failure has happened at the *i* ^th^ sampling time interval, and that the two modes are independent is:
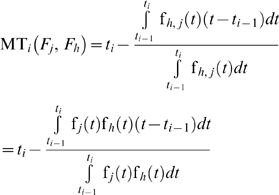
(17)


If the failure time probability density functions of the two failure modes are dependent, we have:
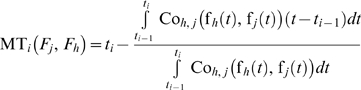
(18)


The expected time from the failure of a system because of the *j* ^th^ and *h* ^th^ failure modes until the time *t_i_*, given that the failure because of the *h* ^th^ failure mode has happened at the *i* ^th^ sampling time interval while the failure because of the *j* ^th^ failure mode has happened before the *i* ^th^ sampling time interval, is:
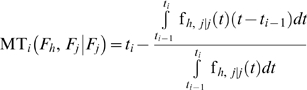
(19)where *f_h,j/h_* is the conditional failure time probability density function of the *h* ^th^ and *j* ^th^ modes given the *j* ^th^ failure mode. If the two modes are independent:
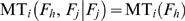
(20)Otherwise, we have
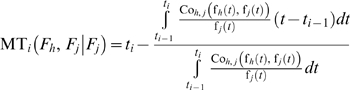
(21)


#### Measurement error functions

Assuming a normal distribution of measurement error due to the *j* ^th^ failure mode with mean *μ_j_* and standard deviation *σ_j_*, the probability density function of the measurement error is:
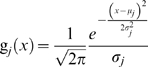
(22)


It is assumed that the probability density function *g*_0_(*x*) of the measurement error of a system during the reliability state is the unit normal distribution (that is *μ*_0_ = 0 and *σ*_0_ = 1).

The probability density function of the so called “intermittent error” is defined as a mixture distribution:
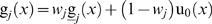
(23)where 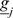 is a normal distribution, *u*_0_ is a uniform distribution with an arbitrary large interval [−ω,ω] and *w_j_* is the probability of the “intermittent error” being operative at a particular moment.

The critical measurement error ce(*x*) is defined as:
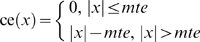
(24)where *x* is the measurement error.

If we denote with *mte* the medically acceptable measurement error, the following critical measurement error measures are defined as the sum of the *d* ^th^ upper and the absolute value of the *d* ^th^ lower partial moments of the measurement error with reference to the *mte* and −*mte* respectively (see *Partial moments* in Appendix S1):
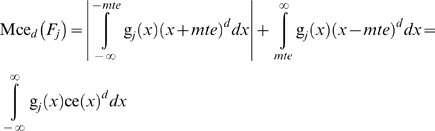
(25)

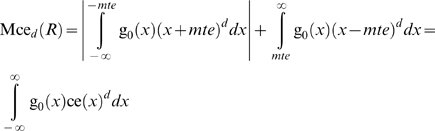
(26)

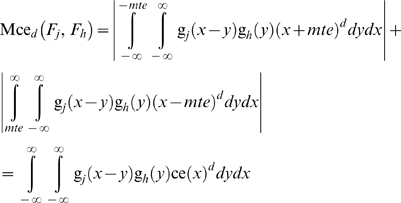
(27)


**Figure 2:**
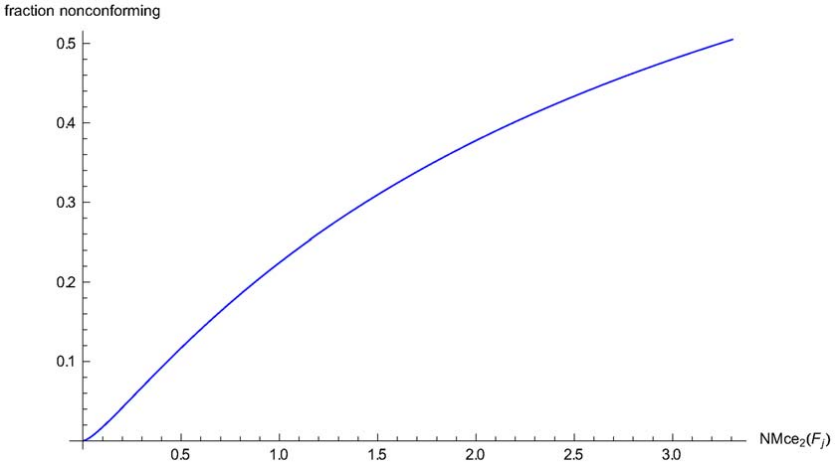
Parametric plot of a critical error measure. Parametric plot of the critical measurement error measure NMce_2_(*F_j_*), based on the normalized sum of the second upper and the absolute value of the second lower partial moments of the measurement error with reference to *mte* and −*mte* respectively, versus the fraction nonconforming, assuming *mte* = 4.0 and a normal measurement error distribution *g_j_*(*x*) with *μ_j_* = 0, and 1≤*σ_j_*≤6.

For *d* = 0 the measures equal the measurement process fraction nonconforming. The normalized the sum of the *d* ^th^ upper and the absolute value of the *d* ^th^ lower partial moments of the measurement error with reference to the *mte* and −*mte* respectively, for any state *S* of the system equals (figure 2):
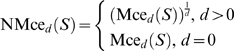
(28)


#### Quality functions

Assuming a QC procedure with a single value QC rule with a decision limit *l*, one control per level and *c* levels of controls with the same measurement error probability density function, the probability of rejection because of the *j* ^th^ failure mode is:
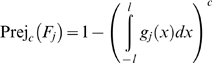
(29)


Assuming an additive measurement error model, the respective probability of rejection because of the combined *j* ^th^ and *h* ^th^ failure modes is:
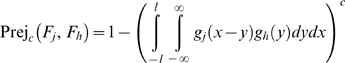
(30)


If the probability density functions of the measurement error of the two failure modes are correlated, the respective probability of rejection because of the combined *j* ^th^ and *h* ^th^ failure modes is:
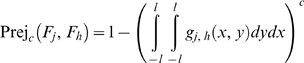
(31)where g*_j,h_*(*x,y*) is a bivariate probability density function (See *Definition of functions assuming multivariate measurement error probability density functions* in Appendix S1).

The probability of rejection of the reliability state is:
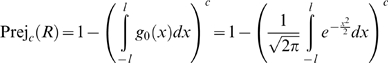
(32)


#### Risk functions

Using eq. (28) we can define the risk function of a state *S*≠*M* that during the *i* ^th^ time interval persists for time *τ_i_* as:
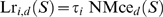
(33)


The residual risk function of the state *S*, assuming one control per level and *c* levels of controls is:
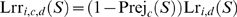
(34)


It is assumed that Lr*_i,d_*(*M*) = 0 and Lrr*_i,,c,d_*(*M*) = 0.

The risk and residual risk are estimated as risk or residual risk per time interval.

The time independent measures
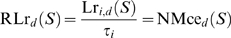
(35)and
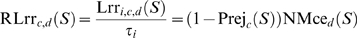
(36)can be considered as the state risk and residual risk rates. These measures can be used instead of the allowable error based quality measures [3]. For *d* = 0 the risk rate equals the measurement process fraction nonconforming, while the residual risk rate equals the fraction nonconforming given the application of the QC procedure. The risk rate can be used for the definition of the critical-failure modes, and the residual risk rate for the definition of the QC procedures.

#### Cost functions

Assuming the application of the QC procedure at one control per level, at *c* levels of controls, the QC related cost functions of the states are defined as:
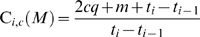
(37)and for *S*≠*M*,
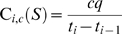
(38)where *q* is the cost of each control and its measurement, and *m* is a maintenance related cost. As cost unit is considered the cost of the analysis of the samples per unit of time in the reliability state.

The QC related cost is estimated as cost per unit of time of each time interval.

### Definitions of the vectors and matrices

It is assumed that the elements of the state vector of an analytical system are the reliability state *R*, the maintenance state *M*, the possible critical-failure modes *F_i_*, and their combinations *F_i_*, *F_j_*,..,*F_n_*. Therefore, the state vector of an analytical system with one failure mode is:
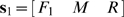
(39)while the state vector of an analytical system with two failure modes is:
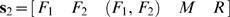
(40)


The respective state probability vectors are:
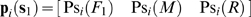
(41)

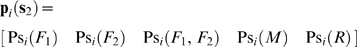
(42)where Ps*_i_*(*S*) the probability of the state *S* at the end of the *i* ^th^ sampling time interval.

If 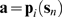 and 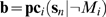, that is the conditional state probability vector given that the system is not in the maintenance state *M* at the end of the *i* ^th^ sampling time interval, where *n* is the number of the elements of the probability state vectors **a** and **b**, and *a*_k_ and *b_k_* the *k* ^th^ element of each vector, then for 1≤*k*≤*n*:
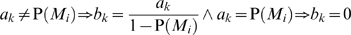
(43)


The state transitions of the systems with one and two failure modes could be respectively presented in matrix form as following:
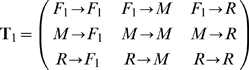
(44)and
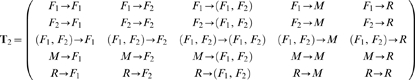
(45)


The **T**_1_ and **T**_2_ are helpful for understanding the definitions of the state transition related matrices.

The state transition probability matrices of systems with one and two failure modes during the *i* ^th^ sampling time interval and before the application of the QC procedure, are respectively (see eqs (6), (9), and (13)):
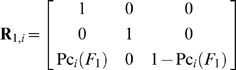
(46)

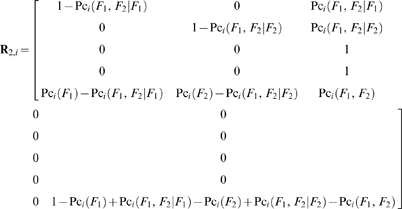
(47)


The state transition probability matrix during the *i* ^th^ sampling time interval and before the application of the QC procedure, of systems with two independent failure modes is:
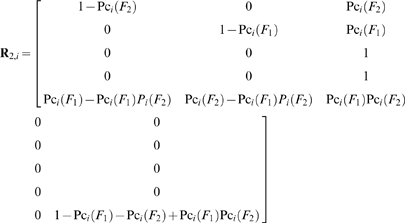
(48)


The matrices of the normalized state mean times from the state transitions during the *i* ^th^ sampling time interval and before the application of the QC procedure, for one and two failure modes, are respectively (see eqs (16) to (20)):
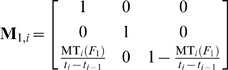
(49)

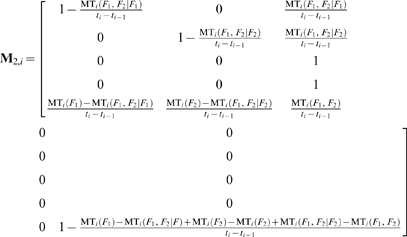
(50)


If the failure modes are independent then:
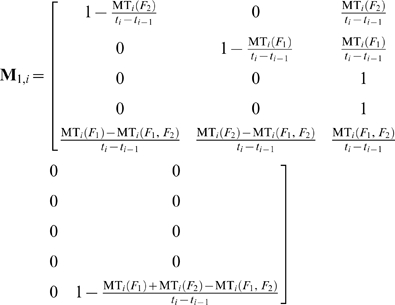
(51)


Assuming one control per level and *c* levels of controls, the state transition probability matrices because of the application of the QC procedure at the end of the *i* ^th^ sampling time interval, of systems with one and two failure states are respectively:
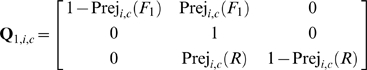
(52)

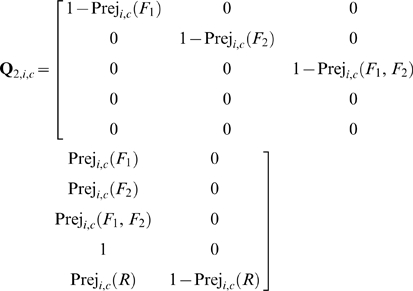
(53)


The risk vectors of systems with one and two failure states are respectively:
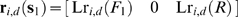
(54)and
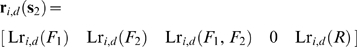
(55)


Assuming one control per level and *c* levels of controls the QC related cost vectors of systems with one and two failure states, during the *i* ^th^ sampling time interval, are respectively:
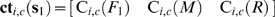
(56)and
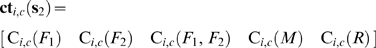
(57)


### Definition of the risk and cost measures

The probabilities of the states at the end of the *i* ^th^ sampling time interval are estimated from:

1. The probabilities of the states at the end of the previous sampling time interval
2. The probabilities of the system critical failures during the sampling time interval.
3. The probabilities of rejection of the states.

The analytical system state probability vector is estimated by the following recursive equation:
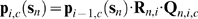
(58)


The risk of the system at the end of the *i* ^th^ sampling time interval is estimated from:

1. The conditional probabilities of the states at the end of the previous sampling time interval, given that the system is not in the maintenance state.
2. The conditional probabilities of the system transition to each state during the *i* ^th^ sampling time interval, given that this transition has not happened during the previous sampling time intervals.
3. The conditional mean times of each state during the *i* ^th^ sampling time interval, given that the state transition has happened during this sampling time interval.
4. The normalized sum of the *d* ^th^ upper and the absolute value of the *d* ^th^ lower partial moments of the measurement error with reference to the *mte* and −*mte* respectively.

The residual risk of the system at the end of the *i* ^th^ sampling time interval is estimated from:

1. The risk of the states of the system at the end of the *i* ^th^ sampling time interval.
2. The probabilities of rejection of the states.

Therefore, the following risk, residual risk, and QC related cost measures are defined respectively as:
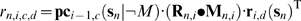
(59)

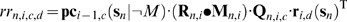
(60)

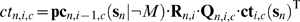
(61)where *n* denotes the number of the failure modes, *i* the *i* ^th^ sampling time interval, *c* the levels of the controls, and *d* the *d* ^th^ upper and lower partial moments of the measurement error with reference to *mte* and −*mte* respectively.

The operator • is the entry wise or Hadamard product operator. Therefore, if 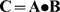, then *c_ij_* = *a_ij_*·*b_ij_*. The operator ^T^ is the transpose operator.

The risk and the residual risk measures of the system are estimated as risk and residual risk per time interval while the expected QC related cost of the system is estimated as cost per unit of time.

### Definition of the measures of the performance of the algorithm

The following measures are used to evaluate the results of the consecutive application of the algorithm, assuming *n* critical-failure modes, one control per level and *c* levels of controls, a risk function based on the normalized sum of the *d* ^th^ upper and the absolute value of the *d* ^th^ lower partial moments of the measurement error with reference to *mte* and −*mte* respectively, initial time *t*_0_ and initial states probability vector **p**(**s** *_n_*):

1. The mean time of the application of the algorithm until the system enters the maintenance state, of a series of up to *k* sampling time intervals, that equals:
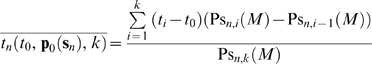
(62)
2. The mean number of sampling time intervals until the system enters the maintenance state, of a series of up to *k* sampling time intervals. This measure is an estimate of the average run length and equals:
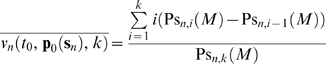
(63)
3. The mean length of the sampling time interval, of a series of up to *k* sampling time intervals that equals:
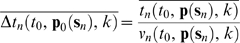
(64)
4. The mean residual risk measure (see eq. (60)) per time interval of a series of up to *k* sampling time intervals that equals:
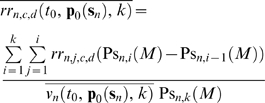
(65)
5. The mean expected QC related cost per time unit measure (see eq. (61)) of a series of up to *k* sampling time intervals that equals:
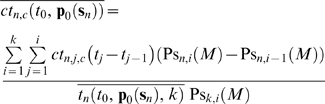
(66)

### About the maintenance state

Although the model can be expanded to include more maintenance related states, as well as a maintenance related state transition probability matrix, it may be applied as it is if we assume that during the maintenance state the system is checked and if there is a critical failure it is repaired. Then the algorithm is applied again, assuming an updated initial states probability vector with the reliability state as the initial state of the system and an updated initial time *t*_0_>0. If the system has been repaired, then the same or revised critical-failure time probability density functions are used. If the system has not been repaired because no critical failure has been found, then the same critical-failure time probability density functions are used.

### Simulations

To explore the mean residual risk per sampling interval and the mean expected QC related cost per time unit using either optimal or predefined QC sampling time intervals I simulated the three analytical systems I to III presented in table 1.

**Table 1:** Parameters of the analytical systems.

Parameter	Notation	Analytical System I	Analytical System II	Analytical System III	
failure modes	*F_j_*	*F_1_*	*F_1_*	*F_1_*	*F_2_*
parameters of the general distribution	*α_j_*	0.5	0.5	0.5	0.5
	*β_j_*	1	1	1	1
	*γ_j_*	0.8	0.9	0.8	0.9
	*θ_j_*	0.001	0.0001	0.001	0.0001
	*λ_j_*	0.001	0.001	0.001	0.001
mean time to failure	MT(F*_j_*)	1956.98	13901.4	1956.98	13901.40
parameters of the error distribution	*μ_j_*	0	0	0	0
	*σ_j_*	3	5	3	5
acceptable measurement error	*mte*	4	4	4	
**acceptable residual risk**	***rr_n,i_***_**,2,2**_	**0.4**	**0.4**	**0.4**	
levels of controls	*c*	2	2	2	
controls per level		1	1	1	
maintenance cost	*m*	4	4	0.4	
QC sample cost	*q*	0.5	0.5	0.05	
moment of risk measures	*d*	2	2	2	
upper bound of risk rate measure	RLr_2_ (*S*)	0.4	0.4	0.4	
upper bound of residual risk rate measure	RLrr_2_,2(*S*)	0.4	0.4	0.4	
QC rules decision limit	*l*	3.18	2.68	2.68	

The parameters of the analytical systems I to III.

The three simulated analytical systems could be clinical chemistry analyzers, measuring for example the serum glucose or cholesterol concentration, with zero bias and coefficient of variation equal to 2.5%, assuming a medically acceptable measurement error equal to 10%.

### Simulations of consecutive applications of the algorithm

#### Relation between residual risk and cost

To explore the relation between the mean residual risk per sampling interval and the mean expected QC related cost per time unit when a QC procedure is applied repeatedly until the system enters the maintenance state, I estimated the mean residual risk 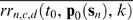 (see eq.(65)) and the mean expected cost measure 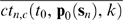 (see eq. (66)) of the analytical systems I to III (see table 1), assuming:

1. Constant length sampling time intervals *Δt_i_* :
  - from *4* to 96 time units, using 4 time units steps,
  - from 8 to 192 time units, using 8 time units steps, and
  - from 4 to 96 time units, using 4 time units steps, respectively.
2. Single value QC rules applied at one control per level, at two levels of controls, with decision limits *l* from 2.0 to 4.0 *σ*_0_ using 0.1 *σ*_0_ steps.
3. The reliability state *R* as the initial state of the systems.
4. Initial times *t*_0_ = 0 and 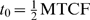, where MTCF is the mean time to critical failure of each analytical system.

To estimate the measures I ran the simulations while Ps*_k_* (*M*)≤0.99, where Ps*_k_* (*M*) is the probability of the maintenance state after the *k* ^th^ consecutive application of the algorithm.

#### Estimation of optimal QC sampling intervals

I ran six illustrative simulations (Ia to IIIb, see table 2), to demonstrate the dynamics of the consecutive application of the algorithm until the system enters the maintenance state. I ran two simulations for each analytical system of the table 1 assuming initial times *t*_0_ = 0 and 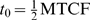 respectively, and the reliability state *R* as the initial state of the systems.

**Table 2:** Statistics of the simulations Ia to IIIb.

	Notation	Simulation Ia	Simulation Ib	Simulation IIa	Simulation IIb	Simulation IIIa	Simulation IIIb
analytical system		**I**	**I**	**II**	**II**	**III**	**III**
initial time	*t*_0_	0	978.49	0	6950.70	0	857.79
mean time until the system enters the maintenance state	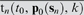	1758.62	1170.14	4529.57	3818.93	1010.45	757.75
mean number of sampling time intervals until the system enters the maintenance state	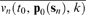	65.22	54.56	51.95	49.15	42.52	39.34
mean sampling time interval length	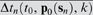	26.96	21.44	87.19	77.69	23.77	19.26
mean residual risk per sampling time interval	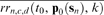	0.400	0.400	0.400	0.400	0.206	0.199
mean expected QC related cost per time unit	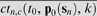	0.053	0.057	0.031	0.031	0.027	0.030

The statistics of the simulations Ia to IIIb. The means were estimated for Ps*_k_* (*M*)≥0.99.

The acceptable residual risk *rr_n,i_*_,2,2_ was set to 0.4 (see eq. 58). The decision limit *l* of the QC rule of each simulation was defined so that RLr*_d_* (*S*)≤0.4 (see eq.(36)) for any state of the respective analytical system. The cost measure to be minimized was the *ct_n,i_*_,2_ (see eq. (61)). To estimate the parameters I ran the simulations while Ps*_k_* (*M*)≤0.99.

For each simulation the measures of the eqs (62) to (66) were estimated.

### Monte Carlo simulations of single application of the algorithm

In addition I ran three Monte Carlo simulations for each analytical system (see table 3), assuming:

**Table 3:** Statistics of the Monte Carlo simulations I to III.

	Notation	Monte Carlo Simulation I	Monte Carlo Simulation II	Monte Carlo Simulation III
analytical system		**I**	**II**	**III**
mean initial time	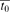	493.19	3502.57	424.79
mean initial state probability vector	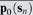	[0.084 0 0.926]	[0.123 0 0.877]	[0.069 0.022 0.002 0 0.907]
mean optimal QC sampling time interval length	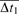	8.29	6.22	20.07
mean residual risk measure of the optimal QC sampling time intervals	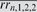	0.400	0.400	0.363
mean expected QC related cost measure of the optimal QC sampling time intervals	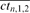	0.165	0.351	0.086

The statistics of the estimated optimal QC sampling time intervals of the Monte Carlo simulations I to III. The means were estimated for 1000 random initial times *t*_0_, with 0≤*t*_0_≤MTCF.

1. Random initial time *t*_0_, 0≤*t*_0_≤MTCF.
2. Initial state probability vector **p**_0_(**s** *_n_*) for each initial time *t*_0_ with (see eqs (41) and (42)):

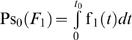
, 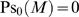, and 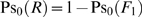, for the Monte Carlo simulations I and II.

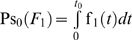
, 
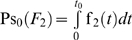
, 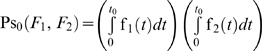, 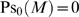, and 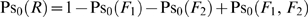, for the Monte Carlo simulation III.

For each analytical system 1000 initial times *t*_0_ were generated. Then for each initial time *t*_0_, after a single application of the algorithm, the residual risk measure *rr_n_*_,1,2,2_ and the expected QC related cost measure *ct_n_*_,1,2_ (see eqs (60) and (61)) were estimated with QC sampling time intervals *Δt*_1_:

- Estimated to be optimal
- With predefined length, from 1 to 120 time units, using 1 time unit steps.

For each initial time *t*_0_ the expected QC related cost per time unit measure of the predefined QC sampling intervals with acceptable residual risk measure was compared with the respective measure of the QC sampling interval estimated to be optimal.

For each predefined QC sampling interval, with acceptable residual risk measures for all the initial times *t*_0_, the mean measure 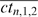 for all the initial times *t*_0_ was compared with the respective mean measure of the QC sampling intervals estimated to be optimal, using the paired Students *t*-test.

## Results

### Simulations of consecutive applications of the algorithm

#### Relation between residual risk and cost

**Figure 3:**
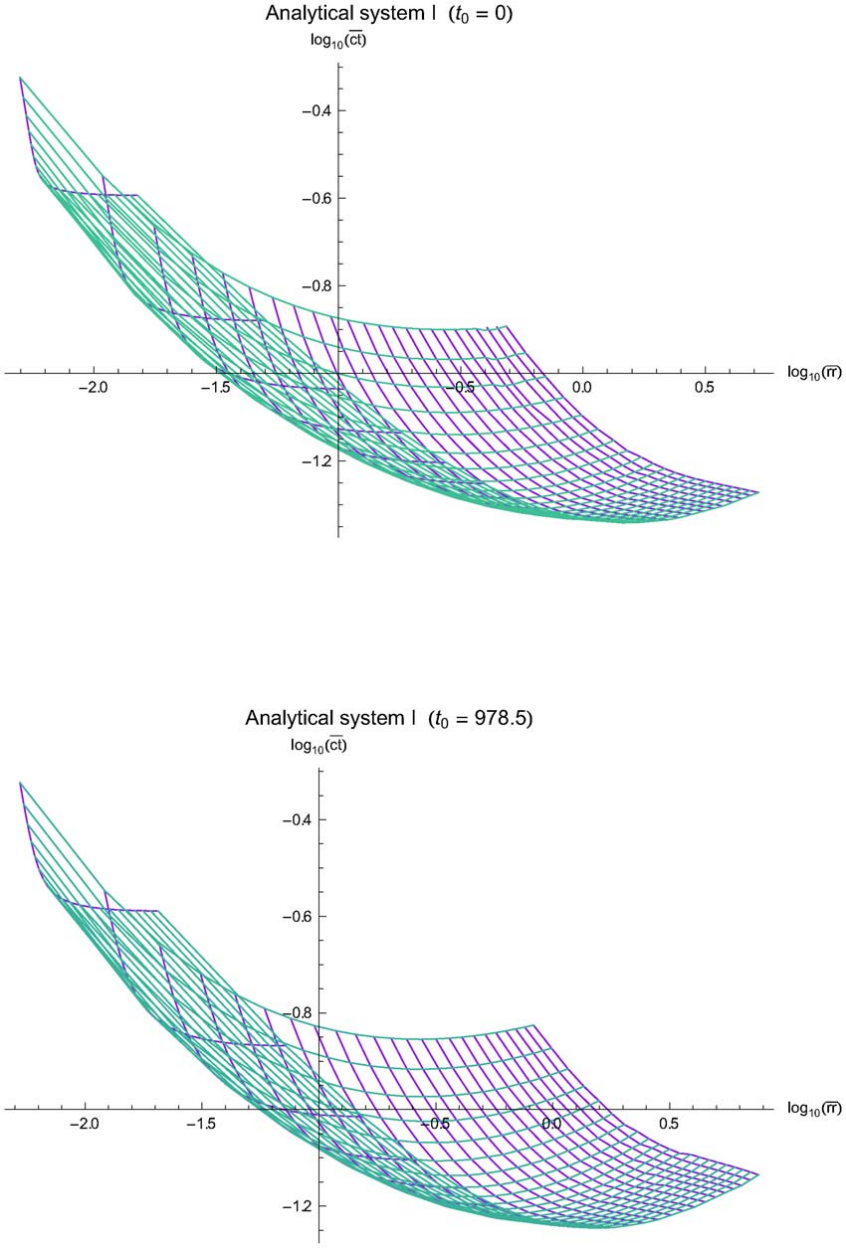
The mean residual risk versus the mean expected QC related cost of the analytical system I. The mean residual risk measure 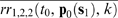 versus the mean expected QC related cost measure 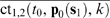 of the analytical system I, assuming initial times *t*_0_ = 0 (upper plot) and *t*_0_ = 978.5 (lower plot), the reliability state as the initial state, constant sampling time interval length *Δt_i_* from *4* to 96 time units, and single value QC rules applied at two levels of controls, with decision limit *l* from 2.0 to 4.0 *σ*_0_. Both means were estimated for Ps*_k_* (*M*)≥0.99. Each purple line joins the points with the same QC sampling time interval length *Δt_i_* and each light blue line joins the points with the same decision limit *l*. The *Δt_i_* is increasing from left to right, while the *l* is increasing from up down.

**Figure 4:**
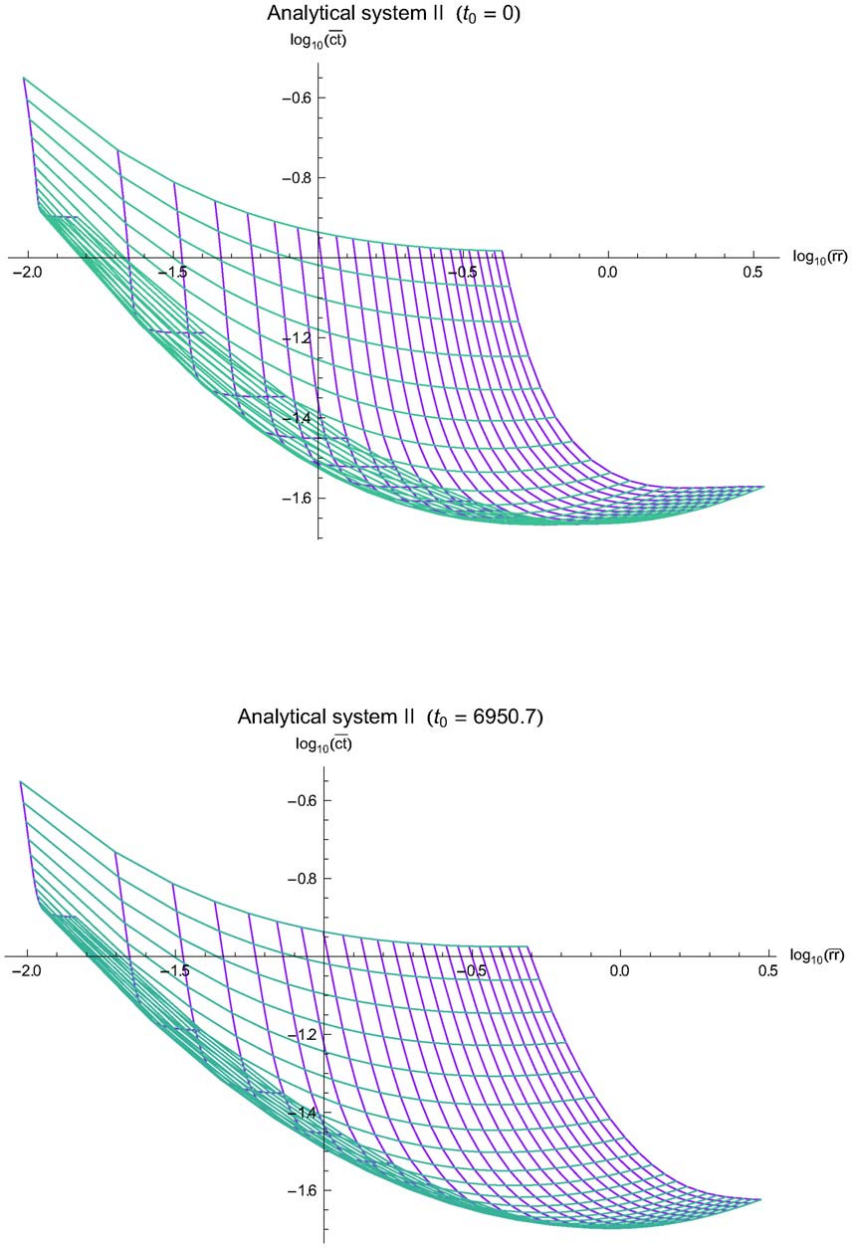
The mean residual risk versus the mean expected QC related cost of the analytical system II. The mean residual risk measure 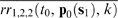 versus the mean QC related cost measure 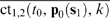 of the analytical system of the simulations II, assuming initial times *t*_0_ = 0 (upper plot) and *t*_0_ = 6950.7 (lower plot), the reliability state as the initial state, constant sampling time interval length *Δt_i_* from 8 to 192 time units, and single value QC rules applied at two levels of controls, with decision limit *l* from 2.0 to 4.0 *σ*_0_. Both means were estimated for Ps*_k_* (*M*)≥0.99. Each purple line joins the points with the same QC sampling time interval length *Δt_i_* and each light blue line joins the points with the same decision limit *l*. The *Δt_i_* is increasing from left to right, while the *l* is increasing from up down.

**Figure 5:**
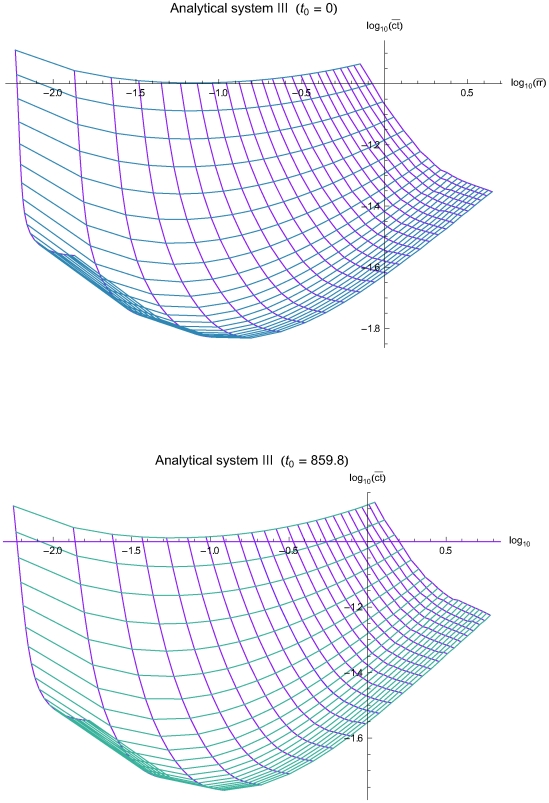
The mean residual risk versus the mean expected QC related cost of the analytical system III. The mean residual risk measure 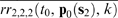 versus the mean QC related cost measure 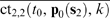 of the analytical system of the simulations III, assuming initial times *t*_0_ = 0 (upper plot) and *t*_0_ = 857.8 (lower plot), the reliability state as the initial state, constant sampling time interval length *Δt_i_* from 4 to 96 time units, and single value QC rules applied at two levels of controls, with decision limit *l* from 2.0 to 4.0 *σ*_0_. Both means were estimated for Ps*_k_* (*M*)≥0.99. Each purple line joins the points with the same QC sampling time interval length *Δt_i_* and each light blue line joins the points with the same decision limit *l*. The *Δt_i_* is increasing from left to right, while the *l* is increasing from up down.

The figures 3, 4 and 5 present the mean residual risk measure 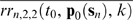 versus the mean expected QC related cost measure 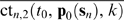 of the analytical systems I to III, assuming initial times *t*_0_ = 0 (upper plots) and 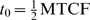 (lower plots). Each purple line joins the points with QC sampling time interval of the same length *Δt_i_* and each light blue line joins the points with the same decision limit *l*. The *Δt_i_* is increasing from left to right, while the *l* is increasing from up down. The relation between the two measures is nonlinear.

#### Estimation of optimal QC sampling intervals

The results of the simulations estimating the optimal QC intervals during the consecutive applications of the algorithm are presented in the table 2 and in the following figures:

1. Simulations Ia (upper plots) and IIa (lower plots): figures 6, 7, and 8.
2. Simulations IIa (upper plots) and IIb (lower plots): figures 9, 10, and 11.
3. Simulations IIIa (upper plots) and IIIb (lower plots): figures 12, 13, 14, and 15.

**Figure 6:**
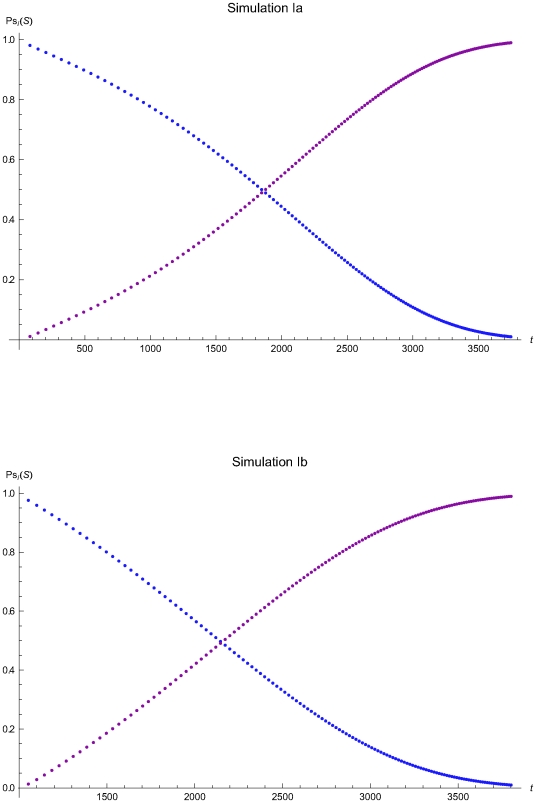
The probabilities of the reliability and maintenance states versus the time of the simulations Ia and Ib. The probabilities of the reliability (*R*) and maintenance (*M*) states versus the time *t* of the simulations Ia (upper plot) and Ib (lower plot), assuming initial times *t*_0_ = 0 and *t*_0_ = 978.5 respectively and the reliability state as the initial state.

**Figure 7:**
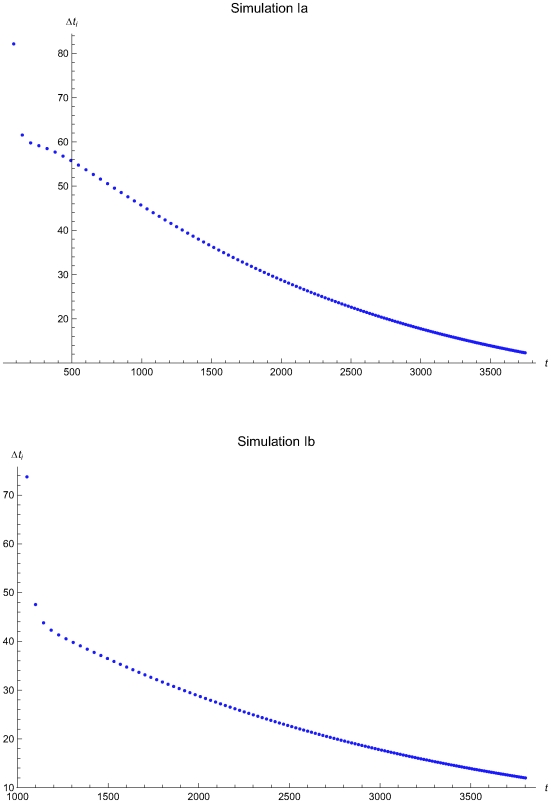
The QC sampling time interval length versus the time of the simulations Ia and Ib. The length of the QC sampling time interval length *Δt_i_* versus the time *t* of the simulations Ia (upper plot) and Ib (lower plot), assuming initial times *t*_0_ = 0 and *t*_0_ = 978.5 respectively and the reliability state as the initial state. The Δ*t_i_* was estimated assuming that the system had not entered the maintenance state for *t*<*t_i−1_*.

**Figure 8:**
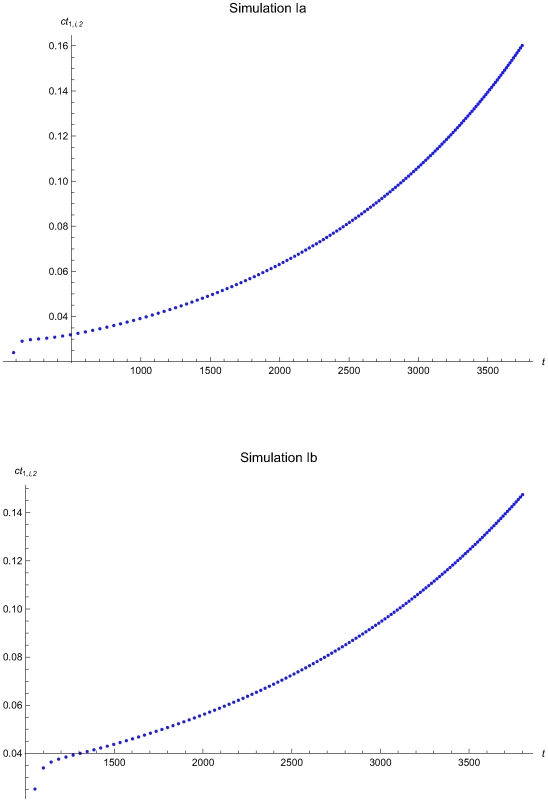
The expected QC related cost versus the time of the simulations Ia and Ib. The expected QC related cost measure ct_1,*i*,2_ versus the time *t* of the simulations Ia (upper plot) and Ib (lower plot), assuming initial times *t*_0_ = 0 and *t*_0_ = 978.5 respectively and the reliability state as the initial state. The ct_1,*i*,2_ was estimated assuming that the system had not entered the maintenance state for *t*<*t_i−1_*.

**Figure 9:**
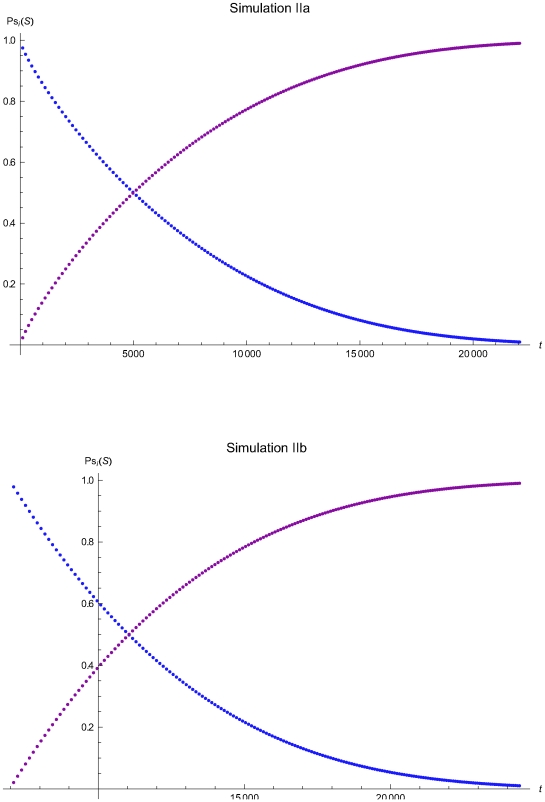
The probabilities of the reliability and maintenance states versus the time of the simulations IIa and IIb. The probabilities of the reliability (*R*) and Maintenance (*M*) states versus the time *t* of the simulations IIa (upper plot) and IIb (lower plot), assuming initial times *t*_0_ = 0 and *t*_0_ = 6950.7 respectively and the reliability state as the initial state.

**Figure 10:**
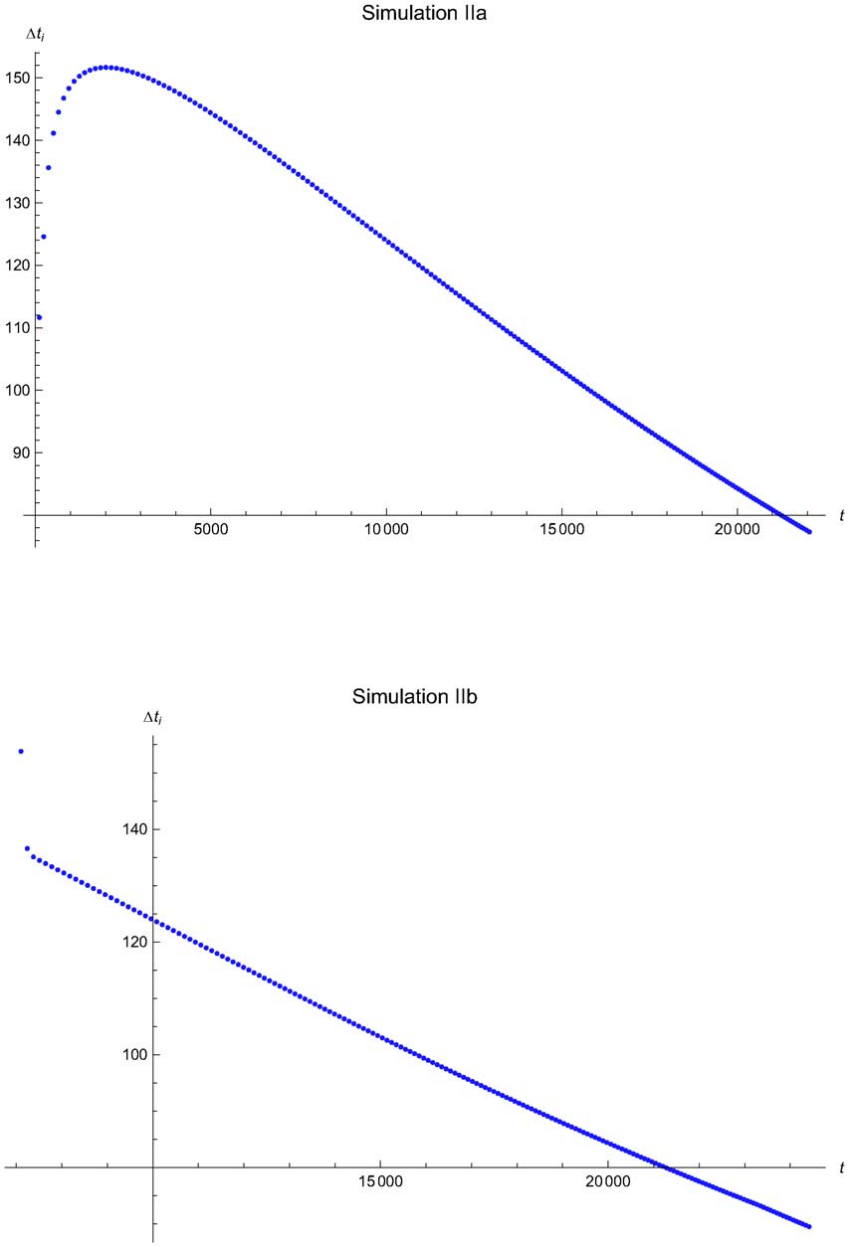
The QC sampling time interval length versus the time of the simulations IIa and IIb. The length of the QC sampling time interval length *Δt_i_* versus the time *t* of the simulations IIa (upper plot) and IIb (lower plot), assuming initial times *t*_0_ = 0 and *t*_0_ = 6950.7 respectively and the reliability state as the initial state. The Δ*t_i_* was estimated assuming that the system had not entered the maintenance state for *t*<*t_i−1_*.

**Figure 11:**
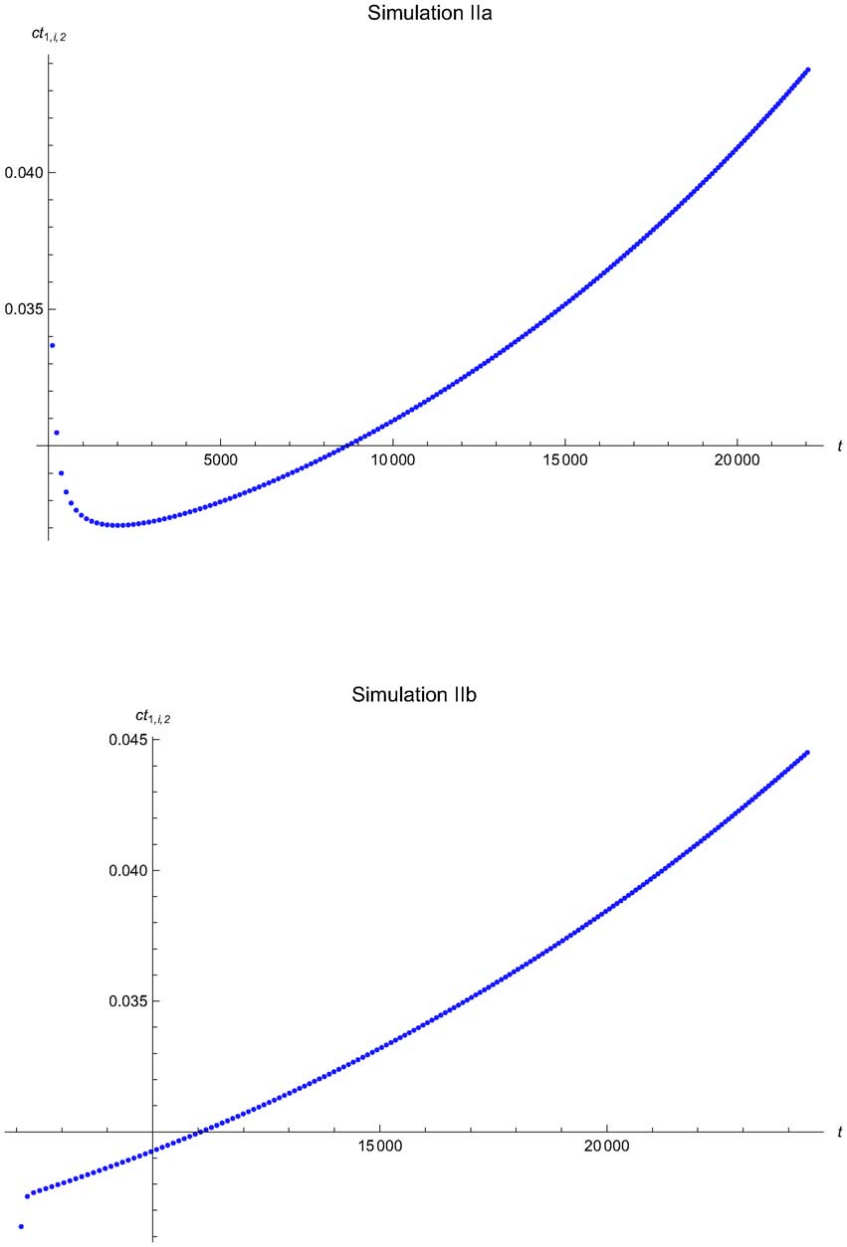
The expected QC related cost versus the time of the simulations IIa and IIb. The expected QC related cost measure ct_1,*i*,2_ versus the time *t* of the simulations IIa (upper plot) and IIb (lower plot), assuming initial times *t*_0_ = 0 and *t*_0_ = 6950.7 respectively and the reliability state as the initial state. The ct_1,*i*,2_ was estimated assuming that the system had not entered the maintenance state for *t*<*t_i−1_*.

**Figure 12:**
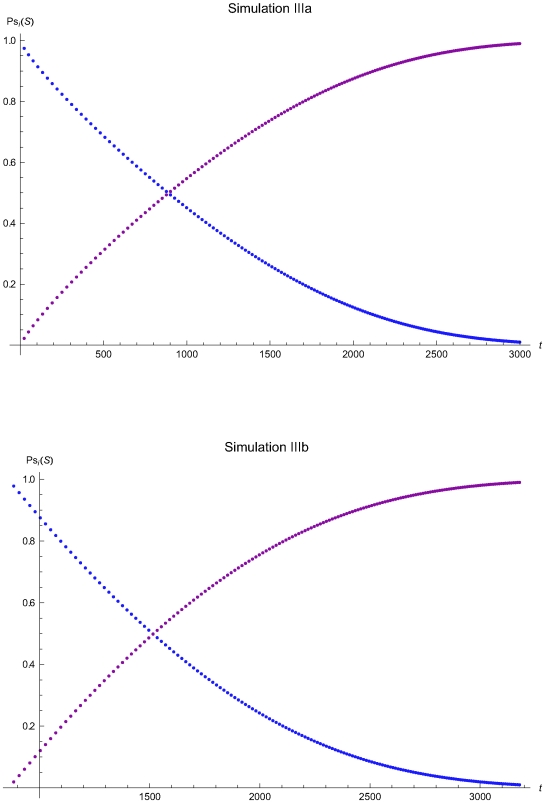
The probabilities of the reliability and maintenance states versus the time of the simulations IIIa and IIIb. The probabilities of the reliability (*R*) and maintenance (*M*) states versus the time *t* of the simulations IIIa (upper plot) and IIIb (lower plot), assuming initial times *t*_0_ = 0 and *t*_0_ = 857.8 respectively and the reliability state as the initial state.

**Figure 13:**
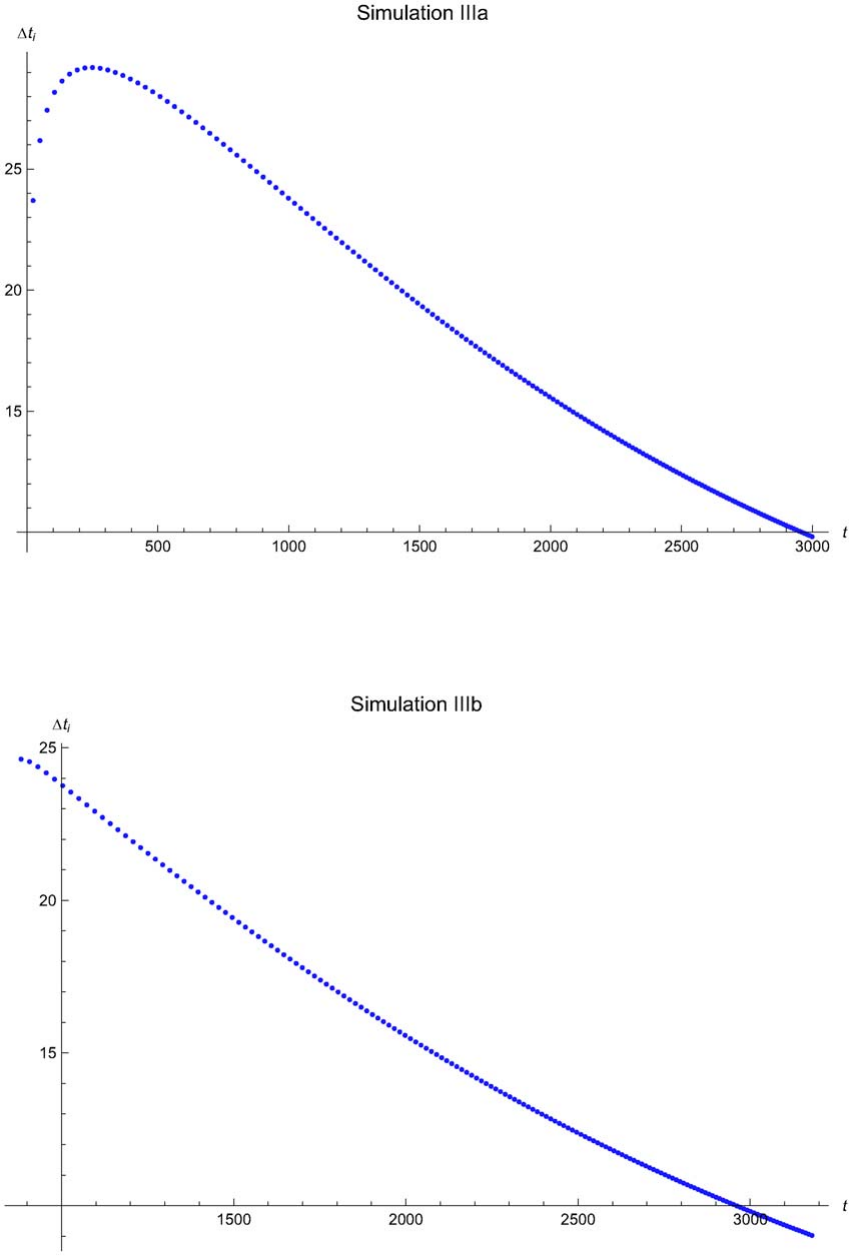
The QC sampling time interval length versus the time of the simulations IIIa and IIIb. The length of the QC sampling time interval length *Δt_i_* versus the time *t* of the simulations IIIa (upper plot) and IIIb (lower plot), assuming initial times *t*_0_ = 0 and *t*_0_ = 857.8 respectively and the reliability state as the initial state. The Δ*t_i_* was estimated assuming that the system had not entered the maintenance state for *t*<*t_i−1_*.

**Figure 14:**
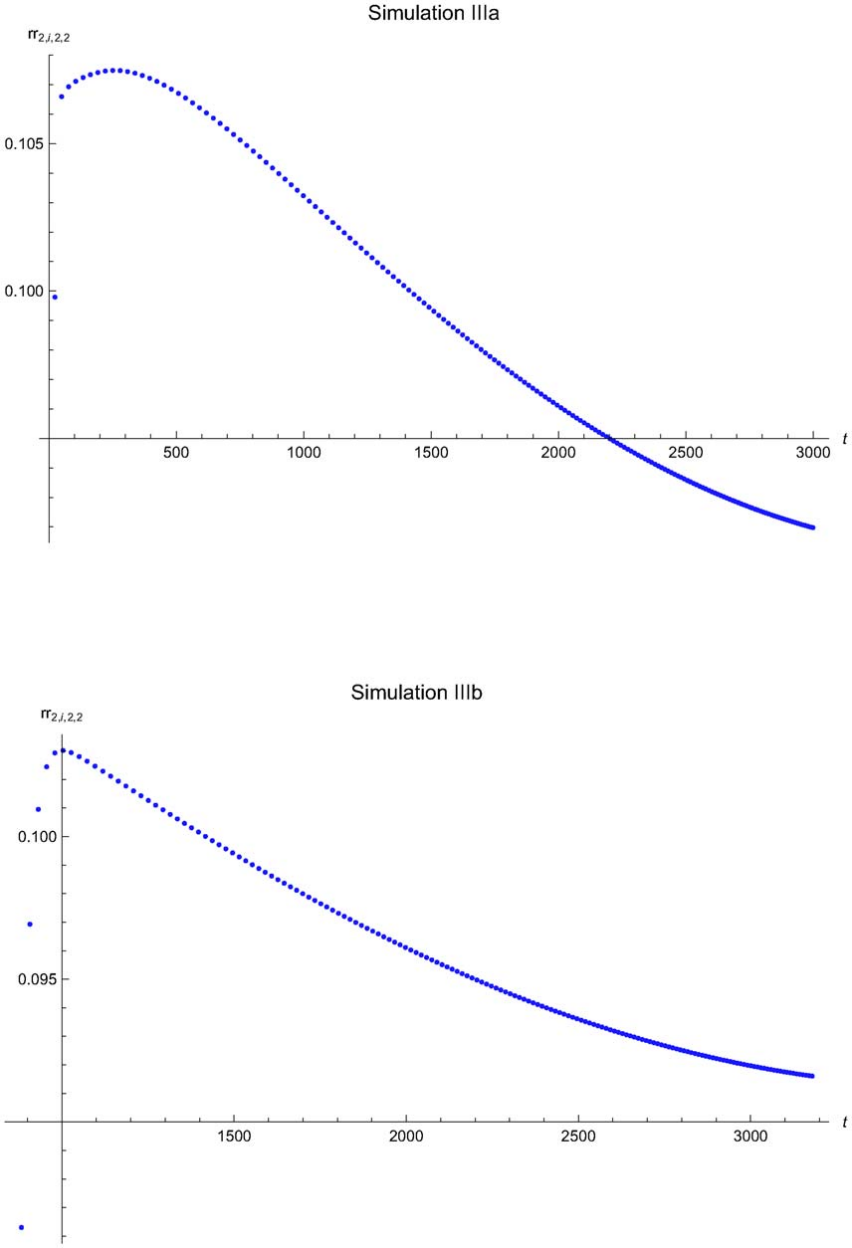
The residual risk versus the time of the simulations IIIa and IIIb. The residual risk measure rr*_2,i_*_,2,2_ versus the time *t* of the simulations IIIa (upper plot) and IIIb (lower plot), assuming initial times *t*_0_ = 0 and *t*_0_ = 857.8 respectively and the reliability state as the initial state. The rr*_2,i_*_,2,2_ was estimated assuming that the system had not entered the maintenance state for *t*<*t_i−1_*.

**Figure 15:**
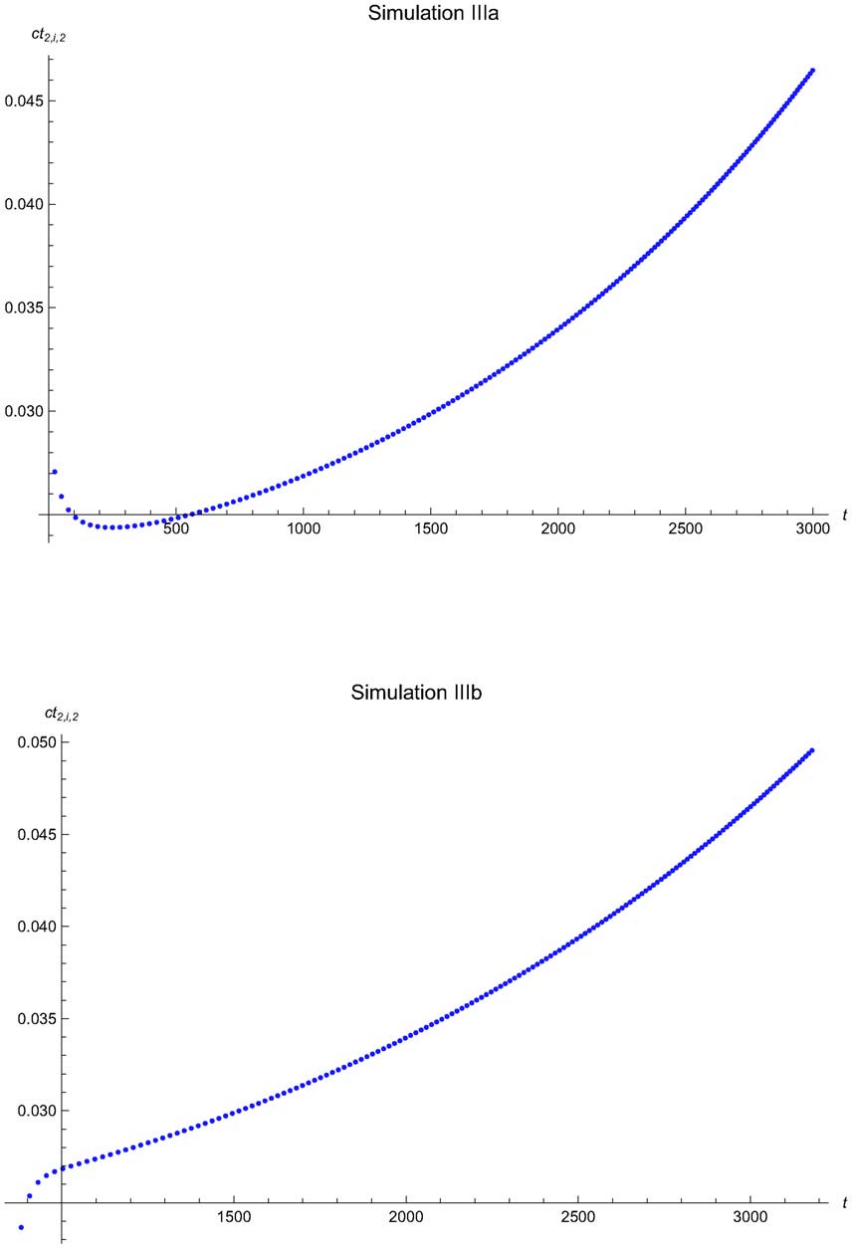
The QC related cost versus the time of the simulations IIIa and IIIb. The QC related cost measure ct_2,*i*,2_ versus the time *t* of the simulations IIIa (upper plot) and IIIb (lower plot), assuming initial times *t*_0_ = 0 and *t*_0_ = 857.8 respectively and the reliability state as the initial state. The ct_2,*i*,2_ was estimated assuming that the system had not entered the maintenance state for *t*<*t_i−1_*.

In all these simulations, an acceptable residual risk was sustained.

The table 2 presents the estimated measures of the eqs (62) to (66) for each simulation.

The figures 6, 9, and 12 present the probabilities of the reliability (*R*) and maintenance (*M*) states versus the time *t* of the simulations Ia, IIa, and IIIa (upper plots) and Ib, IIb, IIIb (lower plots), assuming initial times *t*_0_ = 0 and 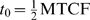 respectively.

The figures 7, 10, and 13 present the length of the QC sampling time interval *Δt_i_* versus the time *t* of the simulations Ia, IIa, and IIIa (upper plots) and Ib, IIb, and IIIb (lower plots), assuming initial times *t*_0_ = 0 and 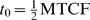 respectively. In all simulations the length the QC sampling time intervals is variable. In general the length of the QC sampling time intervals is decreasing with the time *t*. It is increasing with *t* for 0<*t*≤2032.23 time units in the simulation IIa (see fig. 10) and for 0<*t*≤248.43 time units in the simulation IIIa (see fig. 13).

The figures 8, 11, and 15 present the expected QC related cost measure *ct_n,i_*_,2_ versus the time *t* of the simulations Ia, IIa, and IIIa (upper plots) and Ib, IIb, and IIIb (lower plots), assuming initial times *t*_0_ = 0 and 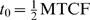 respectively. In general, the ct_1,*i*,2_ is increasing with the time *t*. It is decreasing with *t* for 0<*t*≤2032.23 time units in the simulation IIa (see fig. 11) and for 0<*t*≤248.43 time units in the simulation IIIa (see fig. 15).

The figure 14 presents the residual risk measure *rr_2,i_*_,2,2_ versus the time *t* of the simulations IIIa (upper plot) and IIIb (lower plot), assuming initial times *t*_0_ = 0 and 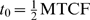 respectively. A remarkable result of these two simulations is that *rr_2,i_*_,2,2_<0.4.

### Monte Carlo simulations of single application of the algorithm

**Figure 16:**
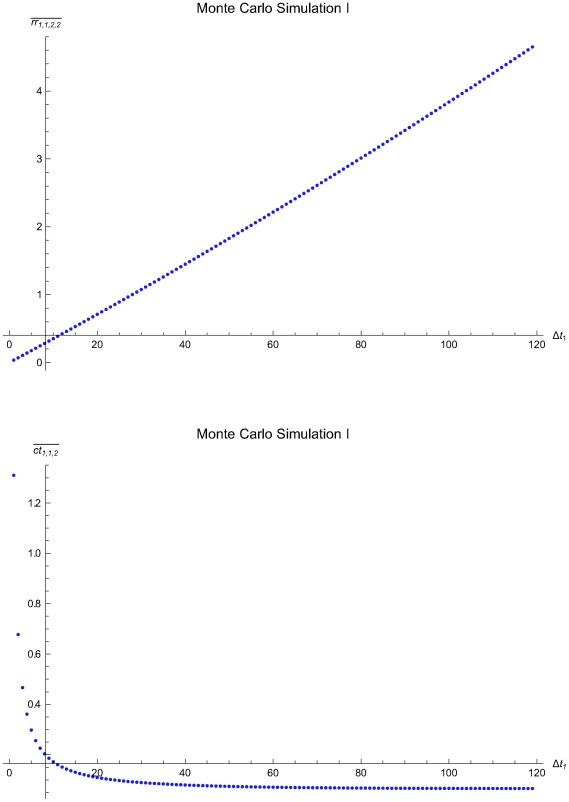
The mean residual risk and QC related cost versus the QC sampling time interval length of the Monte Carlo Simulation I. The mean residual risk measure 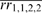 (upper plot) and the mean expected QC related cost measure 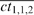 (lower plot) versus the length of the predefined QC sampling time intervals *Δt*_1_ of the of the Monte Carlo Simulation I. The means were estimated after 1000 single applications of the algorithm assuming random initial times *t*_0_, with 0≤*t*_0_≤1957.0. The x-axes origins are set to the mean length of the QC sampling time intervals *Δt_i_* estimated to be optimal. The y-axes origins are set respectively to the mean residual risk measure 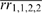 and the mean expected QC related cost measure 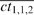 of the QC sampling time intervals estimated to be optimal.

**Figure 17:**
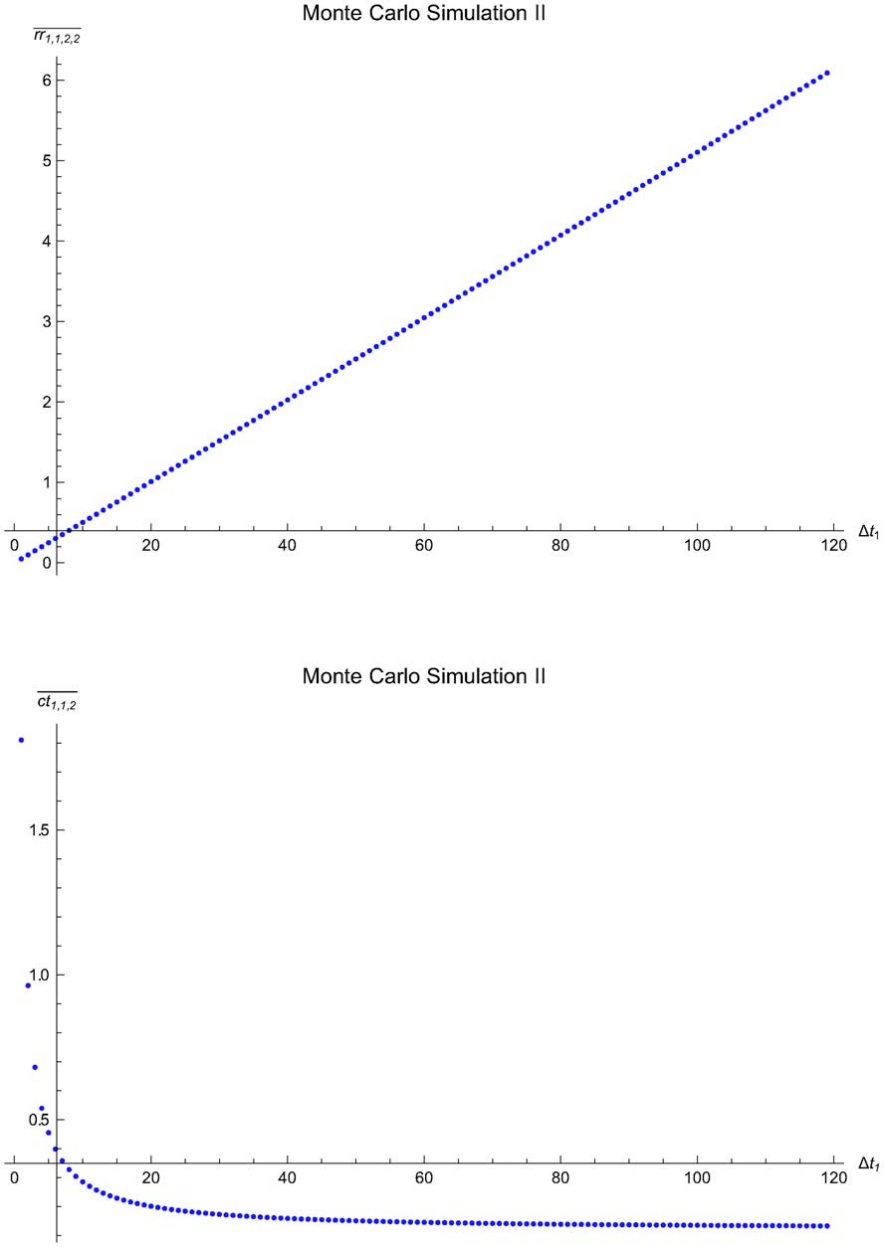
The mean residual risk and QC related cost versus the QC sampling time interval length of the Monte Carlo Simulation II. The mean residual risk measure 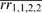 (upper plot) and the mean expected QC related cost measure 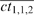 (lower plot) versus the length of the predefined QC sampling time intervals *Δt*_1_ of the of the Monte Carlo Simulation II. The means were estimated after 1000 single applications of the algorithm assuming random initial times *t*_0_, with 0≤*t*_0_≤13901.4. The x-axes origins are set to the mean length of the QC sampling time intervals *Δt_i_* estimated to be optimal. The y-axes origins are set respectively to the mean residual risk measure 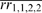 and the mean expected QC related cost measure 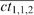 of the QC sampling time intervals estimated to be optimal.

**Figure 18:**
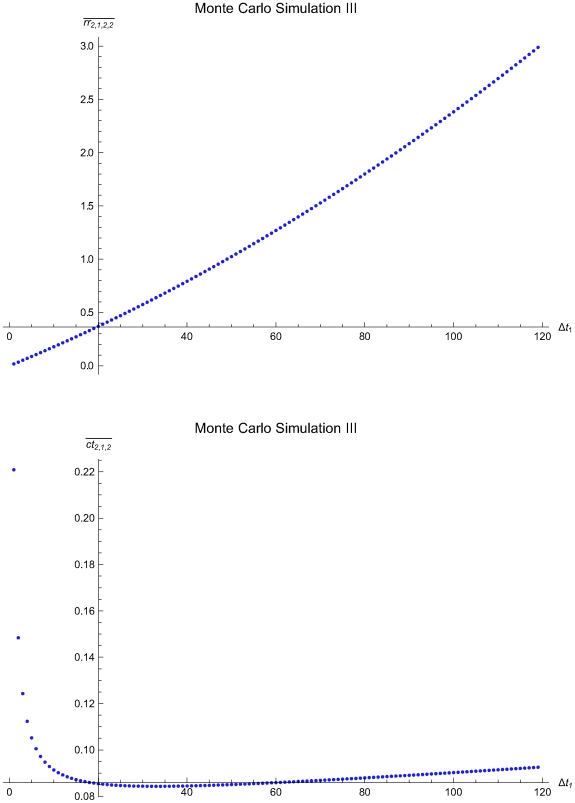
The mean residual risk and QC related cost versus the QC sampling time interval length of the Monte Carlo Simulation III. The mean residual risk measure 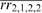 (upper plot) and the mean expected QC related cost measure 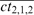 (lower plot) versus the length of the predefined QC sampling time intervals *Δt*_1_ of the of the Monte Carlo Simulation III. The means were estimated after 1000 single applications of the algorithm assuming random initial times *t*_0_, with 0≤*t*_0_≤1715.6. The x-axes origins are set to the mean length of the QC sampling time intervals *Δt_i_* estimated to be optimal. The y-axes origins are set respectively to the mean residual risk measure 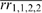 and the mean expected QC related cost measure 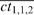 of the QC sampling time intervals estimated to be optimal.

The table 3 presents the parameters of the Monte Carlo Simulations I to III of single application of the algorithm and the statistics of the estimated optimal QC sampling time intervals. The figures 16, 17 and 18 present the mean residual risk measure 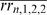 (upper plots) and the mean expected QC related cost measure 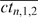 (lower plots) versus the length of the predefined QC sampling time intervals *Δt*_1_ of the Monte Carlo Simulations I to II. The x-axes origins are set to the mean length of the QC sampling time intervals estimated to be optimal. The y-axes origins are set respectively to the mean residual risk measure 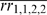 and the mean expected QC related cost measure 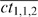 of the QC sampling time intervals estimated to be optimal. The mean residual risk measure 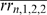 is increasing with the length *Δt*_1_ of the predefined QC sampling time intervals. In general, the mean expected QC related cost measure 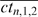 is decreasing with the length *Δt*_1_ of the predefined QC sampling time intervals. It is increasing for 34<*Δt*_1_≤120 time units in the Monte Carlo simulation III.

For each initial time *t*_0_:

1. An optimal QC sampling time interval with acceptable residual risk measure *rr_n_*_,1,2,2_ was estimated.
2. The expected QC related cost measure *ct_n_*_,1,2_ of that interval was less than the respective measure of each predefined QC sampling interval with acceptable residual risk measure.
3. The difference between the length of the predefined QC sampling interval with acceptable residual risk measure *rr_n_*_,1,2,2_ and minimal QC related cost measure *ct_n_*_,1,2_, and the QC sampling time interval estimated to be optimal, was less than one time unit.

The predefined QC sampling intervals *Δt*_1_ with acceptable residual risk measure *rr_n_*_,1,2,2_ for all the initial times *t*_0_ were:

1. From 1 to 5 time units for the Monte Carlo simulation I.
2. From 1 to 4 time units for the Monte Carlo simulation II.
3. From 1 to 11 time units for the Monte Carlo simulation III.

The mean measure 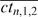 of each of the above predefined QC sampling intervals for all the initial times *t*_0_ was greater than the respective mean measure of the QC sampling intervals estimated to be optimal, with 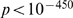. Therefore, in these simulations the proposed method for estimating the QC sampling time intervals outperformed the methods with predefined time intervals.

## Discussion

The algorithm I developed offers an insight in the relationship among a QC procedure, the reliability of an analytical system, the risk of the measurement error and the QC related cost. Furthermore, it demonstrates a method for the rational estimation of the QC sampling time intervals of analytical systems with an arbitrary number of failure modes. Therefore, given the reliability analysis of an analytical system, the risk analysis of the measurement error and a QC procedure, there is an optimal QC sampling time interval approach that can sustain an acceptable residual risk, while minimizes the QC related cost.

The needed quantitative fault tree analysis and the estimation of the critical-failure time probability density functions of the modern analytical systems may be very complex. It is possible though to derive at least upper bounds of them using techniques handling uncertainty [18]. A more complex issue is the estimation of the dependencies between the critical-failure time probability density functions (see eqs (14) and (15)) as well as of the respective measurement error probability density functions (See *Definition of functions assuming multivariate measurement error probability density functions* in Appendix S1). Although the failure time probability density functions of some critical-failure modes of an analytical system may be independent, as for example the failure of an optical component of a photometric module and the purely mechanical failure of a sampling module, others will be dependent. There are techniques that can be used to estimate dependencies [19]. The parametric estimation of multivariate copulas is a method that could be applied [20]. If the dependencies cannot be estimated, then upper and lower bounds of the respective multiple failure time probability density functions can be estimated and interval analysis can be used. For example, we may have:
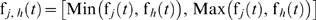
(67)


If the measurement error probability density functions are dependent then multivariate distributions could be used and the respective covariance matrices could be estimated (See *Definition of functions assuming multivariate measurement error probability density functions* in Appendix S1).

This is a large scale procedure that can be accomplished by the industry. Then a database could be established with the reliability analysis data that could be continuously updated with the failure data from the analytical systems in the field, operated by different operators, in different environments. Possibly a substantial commitment is required for such an effort to succeed, giving priority to the safety of the patient.

For the rigorous QC design and estimation of the optimal QC sampling time intervals it is necessary a risk analysis to be performed to correlate the size of the measurement error with the risk that can cause. Then the medically acceptable analytical error, the risk function that can be even a simple step function or a fuzzy function, and the acceptable risk and residual risk measures can be defined. The risk analysis is a very complex task too. It can be subjective or objective, quantitative or semi- quantitative and should be accomplished by the medical profession. In the future, as the potential of the data analysis will increase exponentially, appropriate risk functions should be estimated using evidence based methods.

The currently used QC design methods are based on an upper bound of the fraction of the measurements nonconforming to quality specifications [2]–[4]. I defined the decision levels of the applied QC rules using an approach that although is analogous to these methods, it is more clinically relevant as the risk measures based on the normalized sum of the 2^nd^ upper and the absolute value of the 2^nd^ lower partial moments of the measurement error with reference to *mte* and −*mte* respectively correlate better with the size of the critical error. In addition, I propose a mixture probability density function of the measurement error to model the “intermittent analytical error”.

Preliminary results show that the algorithm I developed can be used to optimize in addition to the QC sampling time intervals the decision limits of the applied QC rules, given the residual risk and the acceptable risk rate RLr*_d_*(*S*) (see eq.(36)). The optimization of both variables though is computationally intensive.

There are numerous assumptions underlying the model I used:

- The critical-failure time probability density functions. I assumed general critical-failure time probability density functions to model a variable hazard rate with a bathtub curve. Any probability density function can be used including the exponential and the lognormal or any mixture distribution. If the failure time probability density functions cannot be estimated, then upper and lower bounds of them can be estimated and interval analysis can be used.
- The assumption of the independence of the failure modes. If the failure modes are dependent the respective dependence functions or at least upper and lower bounds of the respective multiple failure time probability density functions can be estimated.
- The assumption of the normality and additivity of the measurement error. Alternative assumptions can be used.
- The assumption of the same distribution of measurement error of the *c* levels of the controls. For a more general model a multivariate (*c*-variate) distribution of measurement error can be used (See *Definition of functions assuming multivariate measurement error probability density functions* in Appendix S1).
- The QC rules. I applied single value rules but alternative QC rules can be applied as well [16].
- The risk function is based on the normalized sum of the *d* ^th^ upper and the absolute value of the *d* ^th^ lower partial moments of the measurement error with reference to *mte* and −*mte* respectively. Simpler or more complex risk functions can be defined.
- The assumptions about the maintenance state. Alternative assumptions can be used as well.

### Conclusion

To optimize the QC planning process a reliability analysis of the analytical system and a risk analysis of the measurement error are needed. Then it is possible to rationally estimate the optimal QC sampling time intervals to sustain an acceptable residual risk with the minimum QC related cost.

## Supporting Information

Appendix S1 (0.18 MB DOC)
