# Supplementary material for: Estimation of the Optimal Statistical Quality Control Sampling Time Intervals Using a Residual Risk Measure
Source: PLoS One. 2009 Jun 9;4(6):e5770. doi: 10.1371/journal.pone.0005770 (PMC2689359; doi:10.1371/journal.pone.0005770)
Supplement: Appendix S1 — (0.18 MB DOC) [file pone.0005770.s001.doc]

## Notation

### Notation of the states of the system

*R*: The reliability state.

*M*: The maintenance state.

*F*: The failure state.

*F_j_*: The failure state because of the *j*^th^ failure mode.

(*A* | B): The state *A* given the state *B*.

(*A* |¬*B*): The state *A* given the negation of the state *B*.

(*A, B*): The combined states *A* and *B.*

(*A, B |C)*: The combined states *A* and *B,* given the state *C.*

### Notation of the probability functions

P*_i_*(*S*) The probability of the transition to the state *S* during the *i*^th^ sampling time interval.

Pc*_i_*(*S*): The conditional probability of the transition to the state *S* during the *i*^th^ sampling time interval, given that the system was not in that state before *t_i-_*_1_.

Ps*_i_*(*S*): The probability of the state *S* at the end of the *i*^th^ sampling time interval

Prej*_c_*(*S*): The probability of rejection because of the state *S*, assuming one control per level and *c* levels of controls.

### Notation of the probability density functions

g_0_: The probability density function of the measurement error of the reliability state.

g*_j_*: The probability density function of the measurement error because of the *j*^th^ failure mode.

g*_h,j_*: The probability density function of the measurement error because of the *h*^th^ and *j*^th^ failure modes.

$\mathrm{g}_{h,j}^{c}$: The probability density function of the measurement error at the *c*^th^ level of controls because *o*f the *h*^th^ and *j*^th^ failure modes.

$\underline{\mathrm{g}}_{j}$: A component density function of the mixture probability density function of the measurement error because of the *j*^th^ failure mode.

u_0_: A uniform probability density function with an arbitrary large interval [-ω,ω].

f*_j_*: The failure time probability density function of the *j*^th^ failure mode.

f*_h,j_*: The unconditional failure time probability density function of the combined *j*^th^ and *h*^th^ failure modes.

f*_h,j|j_*: The conditional failure time probability density function of the combined *h*^th^ and *j*^th^ failure modes, given the *j*^th^ failure mode.

### Notation of other functions

MTF(*F_j_*): The mean time to failure because of the *j*^th^ failure mode

HR(*F_j_*): The hazard rate of the *j*^th^ failure mode

MTCF: The mean time to critical failure of the analytical system.

Co*_h,j_*(f*_h_*(*t*), f*_j_*(*t*)): A bivariate dependence function of the failure time probability density functions of the *h*^th^ and *j*^th^ failure modes.

MT*_i_*(*S*): The expected time from the transition of a system to the state *S*, assuming that the transition happens during the *i*^th^ sampling time interval.

ce(*x*): The critical error of the measurement error *x*.

Lr*_i,d_*(*S*): The risk function of the *S* state based on the normalized sum of the *d*^th^ upper and lower partial moments of the measurement error with reference to *mte* and –*mte* respectively, for the *i*^th^ sampling time interval.

Lr*_i,d,c_*(*S*): The residual risk function of the *S* state based on the normalized sum of the *d*^th^ upper and lower partial moments of the measurement error with reference to *mte* and –*mte* respectively, for the *i*^th^ sampling time interval, assuming one control per level and *c* levels of controls.

C*_i,c_*(*S*): The cost function of the *S* state, at the end of the *i*^th^ sampling time interval, assuming one control per level and *c* levels of controls.

### Notation of the vectors and matrices

**s***_n_*: The state vector of a system with *n* failure modes.

**p***_i,c_***(s***_n_***)**: The state probability vector of a system with *n* failure modes, at the end of the *i*^th^ sampling time interval, assuming one control per level and *c* levels of controls.

**pc*_i,c_*(*s****_n_*|¬*M***)**: The conditional state probability vector of a system with *n* failure states, at the end of the *i*^th^ sampling time interval, given that the system has not been in the maintenance state, assuming one control per level and *c* levels of controls.

**T***_n_*: The state transition matrix of a system with *n* failure modes.

**R***_n ,i_*: The state transition probability matrix of a system with *n* failure modes at the *i*^th^ sampling time interval.

**M***_n,i_*: The matrix of the normalized expected times from the state transitions of a system with *n* failure modes at the *i*^th^ sampling time interval.

**Q***_n,i,c_*: The transition probability matrix because of the application of the QC procedure of a system with *n* failure modes at the end of the *i*^th^ sampling time interval, assuming one control per level and *c* levels of controls.

**r***_c,d_***(s***_n_***)**: The risk vector of a system with *n* failure modes, assuming one control per level and *c* levels of controls. The risk functions are based on the normalized sum of the *d*^th^ upper and lower partial moments of the measurement error with reference to *mte* and –*mte* respectively.

**ct***_i,c_***(s***_n_***)**: The quality related cost vector of a system with *n* failure states, assuming one control per level and *c* levels of controls.

### Notation of the measures

Mce*_d_*(*S*): A critical error measure of a state *S* based on the *d*^th^ upper and lower partial moments of the measurement error with reference to *mte* and –*mte* respectively.

NMce*_d_*(*S*): A critical error measure of a state *S* based on the normalized sum of the *d*^th^ upper and lower partial moments of the measurement error with reference to *mte* and –*mte* respectively .

RLr*_d_*(*S*): A risk rate measure of a state *S* based on the normalized sum of the *d*^th^ upper and lower partial moments of the measurement error with reference to *mte* and –*mte* respectively .

RLrr*_c_*,*_d_*(*S*): A residual risk rate measure of a state *S* based on the normalized sum of the *d*^th^ upper and lower partial moments of the measurement error with reference to *mte* and –*mte* respectively , assuming one control per level and *c* levels of controls.

*r_n,i,c,d_*: A risk measure of an analytical system with *n* failure modes during the *i*^th^ sampling time interval, based on the normalized sum of the *d*^th^ upper and lower partial moments of the measurement error with reference to *mte* and –*mte* respectively, assuming one control per level and c levels of controls.

*rr_n,i,c,d_*: A residual risk measure of an analytical system with *n* failure modes after the *i*^th^ sampling time interval, based on the normalized sum of the *d*^th^ upper and lower partial moments of the measurement error with reference to *mte* and –*mte* respectively, assuming one control per level and c levels of controls.

$ct_{n,i,c}$ A QC related cost measure of an analytical system with *n* failure modes at the *i*^th^ sampling time interval, assuming one control per level and c levels of controls.

### Notation of the performance measures

$\overline{\mathrm{t}_{n}(t_{0},\mathbf{p}_{0}(\mathbf{s}_{n}),k)}$: The mean total time of application of the algorithm until the system enters the maintenance state, of a series of up to *k* sampling time intervals, assuming *n* critical-failure modes, initial time *t*_0_ and initial state probability vector **p**_0_**(s***_n_***).**

$\overline{v_{n}(t_{0},\mathbf{p}_{0}(\mathbf{s}_{n}),k)}$**:** The mean number of sampling time intervals until the system enters the maintenance state, of a series of up to *k* sampling time intervals, assuming *n* critical-failure modes, initial time *t*_0_ and initial state probability vector **p**_0_**(s***_n_***).**

$\overline{\Delta t_{n}(t_{0},\mathbf{p}_{0}(\mathbf{s}_{n}),k)}$**:** The mean sampling time interval length, until the system enters the maintenance state, of a series of up to *k* sampling time intervals, assuming *n* critical-failure modes, initial time *t*_0_ and initial state probability vector **p**_0_**(s***_n_***).**

$\overline{\mathrm{rr}_{n,c,d}(t_{0},\mathbf{p}_{0}(\mathbf{s}_{n}),k)}$: The mean residual risk measure per time interval, until the system enters the maintenance state, of a series of up to *k* sampling time intervals, assuming *n* critical-failure modes, one control per level and *c* levels of controls, a risk function based on the normalized sum of the *d*^th^ upper and lower partial moments of the measurement error with reference to *mte* and –*mte* respectively, initial time *t*_0_ and initial state probability vector **p**_0_**(s***_n_***).**

$\overline{ct_{n,c}(t_{0},\mathbf{p}_{0}(\mathbf{s}_{n}),k)}$: The mean expected QC related cost per time unit measure, until the system enters the maintenance state, of a series of up to *k* sampling time intervals, assuming *n* critical-failure modes, one control per level and *c* levels of controls, initial time *t*_0_ and initial state probability vector **p**_0_**(s***_n_***).**

### Notation of the parameters

*t_i_*: The end of the *i*^th^ sampling time interval

*l*: The decision limit of the QC rule.

*l_c_*: The decision limit of the QC rule applied to the *c*^th^ level of controls.

*mte*: The medically acceptable measurement error

*μ*_0_: The mean of the measurement error during the reliability state.

*σ*_0_: The standard deviation of the measurement error during the reliability state.

*μ_j_*: The mean of the measurement error because of the *j*^th^ failure mode.

*σ_j_*: The standard deviation of the measurement error because of the *j*^th^ failure mode.

*μ_j,h_*: The mean of the measurement error of the *h*^th^ level of controls because of the *j*^th^ failure mode, assuming a multivariate distribution of the measurement error.

*σ_j,h_*: The standard deviation of the measurement error of the *h*^th^ level of controls because of the *j*^th^ failure mode, assuming a multivariate distribution of the measurement error.

$\underline{\mu}_{j}$: The mean of the component probability density function $\underline{g}_{j}$of the mixture probability density function of the measurement error because of the *j*^th^ failure mode.

$\underline{\sigma}_{j}$: The standard deviation of a component probability density function $\underline{g}_{j}$of the mixture probability density function the measurement error because of the *j*^th^ failure mode

*w_j_*: The probability of the component probability density function $\underline{g}_{j}$of the mixture probability density function of the measurement error because of the *j*^th^ failure mode.

*ρ_j,h,k_*: The correlation coefficient of the measurement error of the *j*^th^ failure mode between the *h*^th^ and *k*^th^ levels of controls, assuming a multivariate distribution of the measurement error.

*ρ_j_*: The correlation coefficient of the measurement error of the *j*^th^ failure mode between the two levels of controls, assuming a bivariate distribution of the measurement error.

*α_j_*: A shape parameter of the general distribution of the *j*^th^ failure mode.

*β_j_*: A shape parameter of the general distribution of the *j*^th^ failure mode.

*γ_j_* : A parameter of the general distribution of the *j*^th^ failure mode.

*θ_j_* : A scale parameter of the general distribution of the *j*^th^ failure mode.

*λ_j_*: A scale parameter of the general distribution of the *j*^th^ failure mode.

*τ_i_*: A time subinterval of the *i*^th^ sampling time interval.

*q*: The cost of each control

*m*: The maintenance state related cost

### Notation of operators

: The dot product

: The Hadamard product

^T^: The transpose operator.

## Partial moments

The *d*^th^ moment of a probability density function g(*x*) of a real variable *x* about a value *c* is defined as:

$$\mathrm{m}(c)_{d}=\int_{-\infty}^{\infty} (x-c)^{d}\mathrm{g}(x)dx$$

The *d*^th^ lower and upper partial moments of the same function g(*x*) with reference to *c* are defined as:

$$\mathrm{m}_{d}^{-}(c)=\int_{-\infty}^{c} (c-x)^{d}\mathrm{g}(x)dx$$

$$\mathrm{m}_{d}^{+}(c)=\int_{c}^{\infty} (x-c)^{d}\mathrm{g}(x)dx$$

For *d* ≥ 0 and *c>0* the normalized sum of the *d*^th^ upper and the absolute value of the *d*^th^ lower partial moments of the measurement error probability density function g(*x*) with reference to  *mte* and –*mte* respectively equals:

$$\mathrm{npm}_{d}(mte)=\left\{\begin{matrix} \left(\left|m_{d}^{-}(-mte)\right|+m_{d}^{+}(mte)\right)^{\frac{1}{d}},d>0 \\ \left(\left|m_{d}^{-}(-mte)\right|+m_{d}^{+}(mte)\right),d=0 \end{matrix}\right.$$

## Definition of functions assuming multivariate measurement error probability density functions

The probability density function of a bivariate normal distribution of the measurement error is:

If the measurement error of the two levels of the concentration of the measurand of the controls is uncorrelated we have:

$$g_{j}(x_{1},x_{2})=\frac{e^{-\frac{1}{2}\left(\left(\frac{x_{1}-\mu_{j,1}}{\sigma_{j,1}}\right)^{2}+\left(\frac{x_{2}-\mu_{j,2}}{\sigma_{j,2}}\right)^{2}\right)}}{2\pi\sigma_{j,1}\sigma_{j,2}}=g_{j,1}(x_{1})g_{j,2}(x_{2})$$

Assuming a bivariate distribution of the measurement error and QC rules applied to one control per level at the two levels of controls, with respective decision limits *l*_1_ and *l*_2_, the probability of rejection because of the *j*^th^ failure mode, at the *i*^th^ sampling time interval is:

$$\mathrm{Prej}_{2}(F_{j})=1-\int_{-l_{2}}^{l_{2}} \int_{-l_{1}}^{l_{1}} \mathrm{g}_{j}\left(x_{1},x_{2}\right)dx_{1}dx_{2}$$

Assuming an additive measurement error model, the respective probability of rejection because of the combined *j*^th^ and *h*^th^ failure modes is:

$$\mathrm{Prej}_{2}(F_{j},F_{h})=1-\int_{-\infty}^{\infty} \int_{-\infty}^{\infty} \int_{-l_{2}}^{l_{2}} \int_{-l_{1}}^{l_{1}} \mathrm{g}_{j}\left(x_{1}-y_{1},x_{2}-y_{2}\right)\mathrm{g}_{h}\left(y_{1},y_{2}\right)dx_{1}dx_{2}dy_{1}dy_{2}$$

The critical measurement error measures that are defined for the *i*^th^ sampling time interval, based on the *d*^th^ upper and the absolute value of the *d*^th^ lower partial moments of the measurement error with reference to *mte* and –*mte* respectively are (see eq. ):

$$\begin{matrix} \mathrm{Mce}_{d}(F_{j})=\frac{\left|\int_{-\infty}^{\infty} \int_{-\infty}^{-mte} \mathrm{g}_{j}\left(x_{1},x_{2}\right)(x_{1}+mte)^{d}dx_{1}dx_{2}\right|+\int_{-\infty}^{\infty} \int_{mte}^{\infty} \mathrm{g}_{j}\left(x_{1},x_{2}\right)(x_{1}-mte)^{d}dx_{1}dx_{2}+\left|\int_{-\infty}^{-mte} \int_{-\infty}^{\infty} \mathrm{g}_{j}\left(x_{1},x_{2}\right)(x_{2}+mte)^{d}dx_{1}dx_{2}\right|+\int_{mte}^{\infty} \int_{-\infty}^{\infty} \mathrm{g}_{j}\left(x_{1},x_{2}\right)(x_{2}-mte)^{d}dx_{1}dx_{2}}{2}= \\ \frac{\int_{-\infty}^{\infty} \int_{-\infty}^{\infty} \mathrm{g}_{j}\left(x_{1},x_{2}\right)\mathrm{ce}(x_{1})^{d}dx_{1}dx_{2}+\int_{-\infty}^{\infty} \int_{-\infty}^{\infty} \mathrm{g}_{j}\left(x_{1},x_{2}\right)\mathrm{ce}(x_{2})^{d}dx_{1}dx_{2}}{2} \end{matrix}$$

$$\begin{matrix} \mathrm{Mce}_{d}(F_{j},F_{h})=\frac{\left|\int_{-\infty}^{\infty} \int_{-\infty}^{\infty} \int_{-\infty}^{\infty} \int_{-\infty}^{-mte} \mathrm{g}_{j}\left(x_{1}-y_{1},x_{2}-y_{2}\right)\mathrm{g}_{h}\left(y_{1},y_{2}\right)(x_{1}+mte)^{d}dx_{1}dx_{2}dy_{1}dy_{2}\right|+\int_{-\infty}^{\infty} \int_{-\infty}^{\infty} \int_{-\infty}^{\infty} \int_{mte}^{\infty} \mathrm{g}_{j}\left(x_{1}-y_{1},x_{2}-y_{2}\right)\mathrm{g}_{h}\left(y_{1},y_{2}\right)(x_{1}-mte)^{d}dx_{1}dx_{2}dy_{1}dy_{2}}{2}+ \\ \frac{\left|\int_{-\infty}^{\infty} \int_{-\infty}^{\infty} \int_{-\infty}^{-mte} \int_{-\infty}^{\infty} \mathrm{g}_{j}\left(x_{1}-y_{1},x_{2}-y_{2}\right)\mathrm{g}_{h}\left(y_{1},y_{2}\right)(x_{2}+te)^{d}dx_{1}dx_{2}dy_{1}dy_{2}\right|+\int_{-\infty}^{\infty} \int_{-\infty}^{\infty} \int_{mte}^{\infty} \int_{-\infty}^{\infty} \mathrm{g}_{j}\left(x_{1}-y_{1},x_{2}-y_{2}\right)\mathrm{g}_{h}\left(y_{1},y_{2}\right)(x_{2}-mte)^{d}dx_{1}dx_{2}dy_{1}dy_{2}}{2}= \\ \frac{\int_{-\infty}^{\infty} \int_{-\infty}^{\infty} \int_{-\infty}^{\infty} \int_{-\infty}^{\infty} \mathrm{g}_{j}\left(x_{1}-y_{1},x_{2}-y_{2}\right)\mathrm{g}_{h}\left(y_{1},y_{2}\right)\mathrm{ce}(x_{1})^{d}dx_{1}dx_{2}dy_{1}dy_{2}+\int_{-\infty}^{\infty} \int_{-\infty}^{\infty} \int_{-\infty}^{\infty} \int_{-\infty}^{\infty} \mathrm{g}_{j}\left(x_{1}-y_{1},x_{2}-y_{2}\right)\mathrm{g}_{h}\left(y_{1},y_{2}\right)\mathrm{ce}(x_{2})^{d}dx_{1}dx_{2}dy_{1}dy_{2}}{2} \end{matrix}$$

If the measurement error of the two failure modes is correlated then a quad-variate pdf can be used. If the measurement error of the two levels of the concentration of the controls is uncorrelated then we have:

$$g_{j,h}(x_{j,1},x_{j,2},x_{h,1},x_{h,2})=g_{j,h}^{1}(x_{j,1},x_{h,1})g_{j,h}^{2}(x_{j,2},x_{h,2})$$

where $g_{j,h}^{1}$and $g_{j,h}^{2}$ the probability density functions of the measurement error at the 1^st^ and 2^nd^ level of the controls because of the the  *j*^th^ and *h*^th^ failure modes.

In general, the probability density function of the measurement error of the *c* correlated levels of the concentration of the measurand of the controls because of *n* critical-failure modes with correlated measurement error is *cn*-variate. The respective covariance matrix can be estimated [21-23].
